# Understanding spatiotemporal variation of heatwave projections across US cities

**DOI:** 10.1038/s41598-025-95097-5

**Published:** 2025-03-27

**Authors:** Saurav Bhattarai, Laxman Bokati, Sanjib Sharma, Rocky Talchabhadel

**Affiliations:** 1 Department of Civil and Environmental Engineering, Jackson State University, Jackson, MS USA; 2 School of Sustainable Engineering and the Built Environment, Arizona State University, Tempe, AZ USA; 3 Department of Civil and Environmental Engineering, Howard University, Washington, DC USA

**Keywords:** Heatwaves, Shared socioeconomic pathways, Climate change, Vulnerability, Climate and Earth system modelling, Climate-change impacts, Projection and prediction

## Abstract

Heatwaves are one of the deadliest meteorological hazards, posing a substantial risk to human health, environment, and the economy. The frequency and intensity of heatwaves have substantially escalated throughout the United States (US), as evidenced by a noticeable contrast between the average occurrence of two heatwaves per year during the 1960s and the surge to six per year during the 2010s. The primary objective of this study is to assess the impacts of past heatwave events in major US cities, project future scenarios, and analyze the relationship between heatwaves and their associated health and environmental consequences. First, we investigate the spatio-temporal variations in the intensity, frequency, and duration of past heatwaves, along with anticipated changes under various shared socioeconomic pathways till the end of 2100. Secondly, we examine the adverse heatwave impacts on human health and well-being, considering both current demographics and future projections. Our results highlight significant projected increases in heatwave frequency, intensity, and duration across all US regions, with the most dramatic escalations under high-emission scenarios. By the late twenty-first century, cities could experience up to 2–4 times the frequency and duration of heatwave days annually compared to the baseline period (1985–2014), with night-time heatwave durations potentially covering over half the year in some regions. Furthermore, analysis using composite heatwave indices reveals severe heat stresses, particularly in southwestern cities like Las Vegas and Yuma, and southern cities like Miami, indicating a rising trend of heatwave susceptibility due to both climatic and demographic shifts. This study contributes to the growing body of research advocating proactive measures to address the escalating threat of heatwaves. By integrating projected climate indices and demographic shift, it provides a nuanced assessment of urban heatwave vulnerability, with a specific focus on densely populated cities and high-risk regions.

**Supplementary Information:**

The online version contains supplementary material available at 10.1038/s41598-025-95097-5.

## Introduction

Record-shattering temperature extremes profoundly affect both the environment and society^1–3^. Historical observations and model projections depict that the worst heatwaves are still on the horizon due to both natural factors and anthropogenic climate change^4–6^. There is a noticeable rise in the frequency and intensity of heatwaves across the globe^7,8^. These events, characterized by prolonged periods of excessively high temperatures^9,10^, have become a serious concern, particularly in densely populated urban cities^11–13^. The phenomenon is not confined to inland; the oceans too experience heatwaves, affecting marine ecosystems and weather patterns far beyond their immediate vicinity^8,14^.

Urban centers, with their high population densities and extensive infrastructure^15,16^, are especially vulnerable to the adverse effects of these temperature extremities^17,18^. In addition, the built environment (generally impervious and heat absorbing) in cities exacerbates the situation^17,19,20^. Heatwave consequences are far-reaching, affecting both the economy and the social fabric of urban communities^21–23^. Economically, the demand for energy skyrockets as individuals and businesses seek to cool down, placing a significant strain on power grids and leading to increased energy costs^24–26^. Socially, the health risks associated with extreme heat disproportionately affect the most vulnerable members of society, including the elderly, children, and those with pre-existing health conditions^12,27–29^. The phenomenon of urban heat islands, where city temperatures can be significantly higher than surrounding rural areas due to human activities, further intensifies these effects^30,31^. This can lead to surface temperature differences as high as 10 °F within the same metropolitan area, amplifying the health and economic impacts of heatwaves on urban populations^32–34^. In fact, surface temperature pattern reveals intra-urban temperatures can be as large as, or even larger than, urban-rural differences^32^.

Several previous studies highlighted the catastrophic consequences of heatwaves in multisector dynamics^35–37^, significantly impacting the economy and exacerbating socioeconomic disparities^22,38–41^. For instance, the 2012 heatwave in the Northeast US demonstrates how vulnerable urban infrastructures and the economy are to sudden climatic shifts, highlighting the pressing need for adaptive strategies to mitigate future risks^42^. Similarly, in southern California, the persistence of heatwaves has threatened vital ecosystem services crucial for local economies and biodiversity^43^. Moreover, the disproportionate effects of climate impacts on marginalized communities in the US reveal deep-seated inequalities, necessitating targeted interventions to ensure equitable resilience building^35,37,44,45^. This inequality is manifested in increased health risks, loss of productivity, and heightened energy costs, laying bare the socioeconomic fault lines exacerbated by heatwaves^46,47^. As climate change continues to intensify, the economic burden of heatwaves is expected to escalate^48,49^, calling for comprehensive policies that address both immediate and long-term challenges posed by these extreme weather events^50–52^. These heatwaves pose serious and escalating threat to urban resilience, impacting critical infrastructures, public health and economic stability^11,38,53^. There is an urgent need of comprehensive strategies to mitigate the impacts of heatwaves, particularly in urban settings where the concentration of people and infrastructure amplifies the risks associated with these extreme temperature events^24,54–56^.

Despite the growing awareness of the risks posed by heatwaves, there is still a considerable gap in our understanding of their full impacts across a range of spatiotemporal scales, how risk will evolve under dynamic environmental and socioeconomic conditions, and potential mitigation strategies in densely populated cities. While previous studies have examined the growing heatwave characteristics in the major urban areas, they have primarily focused on daytime heatwaves within a historical time frame^57,58^. However, the significance of nighttime heatwaves, their associated health impacts, and the additional energy usage required to mitigate them in metropolitan areas has not been extensively explored. In addition, the projection of this growing concern is not done at these places with dense populations, which is crucial for planning effective adaptation and mitigation measures.

Building on our previous analysis of heatwave exposure in Mississippi, which utilized historical data to characterize heatwave patterns across vulnerable communities^29^, this study expands the scope to (i) assess a broader set of urban areas using both historical and projected climate data, (ii) quantify heatwave risk by integrating future projections of population and heatwaves to assess urban vulnerability, (iii) identify the hotspots in both coastal and inland cities. This continuation allows us to evaluate the potential future impacts of heatwaves under various climate scenarios, providing a more comprehensive view of urban heat risks. By investigating the spatial and temporal patterns of heatwaves and their implications for human health and urban ecosystems, this study seeks to provide valuable insights into effective strategies for enhancing urban resilience to climate extremes. This, in turn, can inform policy development that can help protect vulnerable populations and ensure the sustainability of urban environments in the face of increasing climatic challenges.

## Materials and methods

### Study area

**Fig. 1:**
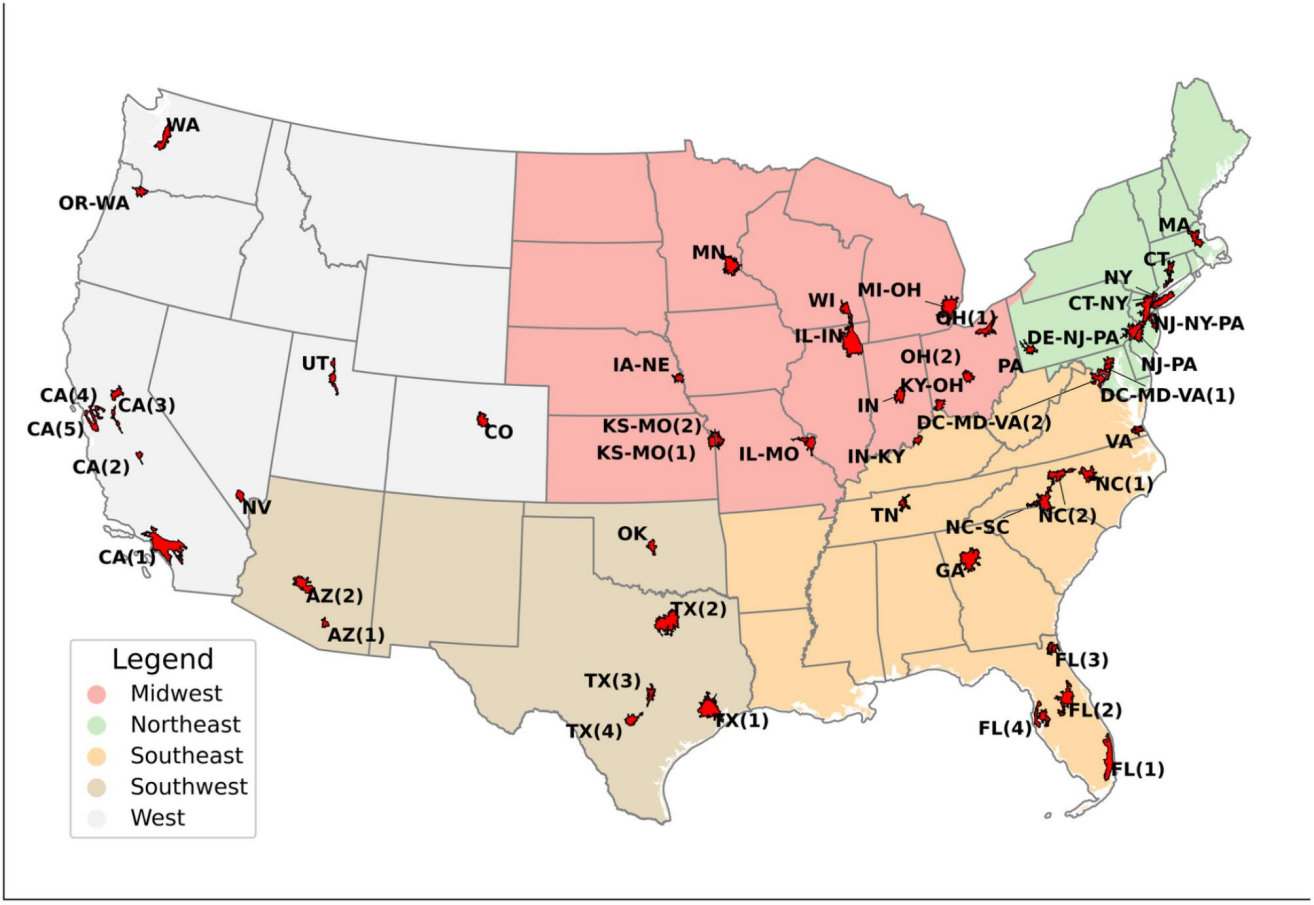
Top 50 most populous US Cities grouped by region. The text on the map represents a different City ID. The name of the city associated with each city ID is listed in Table S1 in the supplementary section. Different colors represent different regions divided by state boundaries.

We focus our analysis on the 50 most populous US cities where frequent and damaging heatwaves have posed serious concerns. Selection of these cities involves a multi-step workflow. First, over 1000 global cities are acquired from the open repository^59^. While the US Census identifies over 10,000 individual cities^60^, many of these represent adjacent municipalities that share similar climate characteristics. Therefore, this global inventory consolidates nearby cities into unified urban areas (> 100 km^2^), yielding 247 distinct urban regions within the contiguous US. Using population projection grids (at 1 km spatial resolution) under five SSPs^61^, we ranked these 247 urban areas by 2020 population and selected the top 50 most populous regions for our analysis. As depicted in Fig. 1, these 50 cities are distributed across the country across varying climates. Table S1 in the supplementary section provides detailed information, including unique ID, and city names. We additionally define five broader regions (namely, Northeast, Southeast, Midwest, Southwest, West) by grouping neighboring cities.

### Data collection

We collect both historical and projected climate data. Historical data are used to analyze past heatwave trends. Global climate model projections are used to analyze future risk of heatwaves.

### CMIP6 global climate model projections

We utilized the NASA Global Daily Downscaled Projections (NASA GDDP-CMIP6) dataset, which provides bias-corrected and downscaled daily climate projections at 0.25° × 0.25° (~ 27.83 km) spatial resolution. The downscaling process employs the Bias-Correction Spatial Disaggregation (BCSD) method, which first removes systematic biases in the GCM outputs using quantile mapping against observational data, then applies spatial disaggregation to achieve higher resolution. This dataset was specifically chosen for its comprehensive coverage, robust downscaling methodology, and wide adoption in climate impact studies^62^. Downscaled projections (at ~ 27.83 km spatial resolution) from 29 global climate models^62^ under two shared socioeconomic pathways: SSP2-4.5 and SSP5-8.5 are used in this study. Specifically, we acquire the daily minimum (*tasmin*) and maximum (*tasmax*) near-surface air temperatures for historical (baseline: 1985–2014), near future (NF: 2025–2054) and far future (FF: 2065–2094) periods. The SSP2-4.5 represents an intermediate greenhouse gas emissions scenario, consistent with policies and technologies that gradually reduce emissions. Global warming reaches 2.7 °C above pre-industrial levels by 2100 under SSP2-4.5. On the other hand, SSP5-8.5 corresponds to a ‘fossil-fueled development’ future with high greenhouse gas emissions in the absence of climate policies. This pathway leads to around 4.5 °C of global warming by 2100 ^63^. SSP2-4.5 and SSP5-8.5 are chosen to capture a range of plausible climate futures and heatwave risks, representing moderate and high-end greenhouse gas emissions scenarios, respectively^64^. Analyzing both pathways allows us to quantify heatwave risks across diverging climate futures. While our analysis includes both ESMs and traditional coupled atmosphere-ocean models, ESMs are more heavily represented in our dataset (Table S2). Future work could explicitly compare the performance of ESMs versus traditional coupled models in representing urban climate, particularly as it relates to heat extremes.

### Re-analyzed ERA5 data

In addition to future projections, we collect daily average 2 m air temperature data from the ERA5 reanalysis dataset (~ 11.13 km spatial resolution) for the baseline period. ERA5 provides consistent and spatially complete global climate data by assimilating both model estimates and ground-based observations^65^. ERA5 temperature data enables validation of the climate models during the historical period and helps evaluate how well the selected climate models are able to mimic observed heatwave trends and variability. While ERA5 provides comprehensive spatial coverage and temporal consistency, we acknowledge its limitations in fully capturing urban-scale temperature variations. Previous studies^66^ have shown that ERA5 tends to underestimate urban heat island effects due to its spatial resolution. However, ERA5 has demonstrated good agreement with station observations for detecting heatwave events and capturing temperature extremes at regional scale^65,67^. To adress potential urban-scale biases, we examine the ERA5 data alongside ground-based observations. For each city, we identify nearby stations using the Meteostat Python library^68^ and compare the datasets using R² to assess the reliability of ERA5 for validation purposes.

### Gridded population data

We obtain 1 km resolution gridded population projections from 2020 to 2100 under the five SSPs to map heatwave exposure onto varying socioeconomic futures^61^. Overlaying the projected extreme heat patterns onto spatial population grids under different SSPs, we identify locations expected to experience both high heatwave intensity and social vulnerability. These SSPs represent varying socioeconomic futures and policy assumptions^69^, including: SSP1 (Sustainability: high investments in education and health, low population growth), SSP2 (Middle of the Road: continuation of current trends), SSP3 (Regional Rivalry: growing resource intensity and population, barriers to trade), SSP4 (Inequality: unequal investments focused on elites), SSP5 (Fossil-fueled Development: rapid economic growth and energy intensification). Overlaying projected heat waves onto population maps across these varying futures, we then quantify exposure risks and adaptive capacities stemming from socioeconomic factors.

### Dataset preprocessing

Multiple daily temperature datasets are derived from the acquired raw climate variables to support identification and characterization of historical and projected heatwave events over varying diurnal and multi-decadal periods. Daytime Extreme Heat Dataset is derived from *tasmax*, capturing only daytime heatwave events defined as 3 + consecutive days exceeding the 95th percentile *tasmax* threshold, and Nighttime Extreme Heat Dataset is constructed identically but utilizing *tasmin* as the input to delineate overnight heatwaves. The 95th percentile temperature thresholds were calculated using the baseline period (1985–2014) for each city and were held constant when analyzing near future (2025–2054) and far future (2065–2094) periods. This approach enables direct comparison of future conditions against historical baselines, allowing us to quantify changes in heatwave frequency, intensity, and duration relative to past temperature norms. While humidity-based metrics like Heat Index and Wet Bulb Temperature provide important information about heat stress, this study focused on temperature-based indices to ensure robust model evaluation and maintain consistency across different data sources. Future studies could explore incorporating humidity metrics as CMIP6 humidity projections become more refined.

### Computation of heatwave indices individually and regionally

Leveraging the multi-dimensional temperature datasets, various heatwave indices are computed to characterize intensity, frequency, and duration at the city and regional scales across historical and future time periods. The following metrics in Table 1 are derived individually for each of the 50 cities as well as five broader regions encompassing groups of cities:

**Table 1:** Heatwave metrics used in this study.

SN	Metrics	Definition	Attributes
1	Heatwave number (HWN)	Annual count of events with duration ≥ 3 days and temperature > 95th percentile.	Frequency
2	Heatwave total days (HWTD)	Sum of days per year within heatwaves	Duration
3	Heatwave longest duration (HWLD)	Maximum consecutive days in a single heatwave at a year	Peak duration
4	Heatwave mean temperature (HWMT)	Mean temperature across all heatwave days per year	Intensity
5	Hottest heatwave temperature (HHT)	Highest single-day maximum temperature recorded during any heatwave in a year	Extreme peak intensity

### Climate model evaluation

We compute the mean values of the heatwave indices for each city from both the ERA5 dataset and the 29 global climate models. We quantify the climate model deviation from observation by calculating the absolute differences between the ERA5-derived indices and those obtained from the climate models. The absolute differences serve as the basis for ranking climate models. For each city, we rank the models for each heatwave index (HWN, HWTD, HTLD, HWMT, and HHT). These rankings are then averaged to derive a single, comprehensive ranking for each model’s performance in simulating heatwave events across the cities. To synthesize the rankings into a coherent analysis, we assign weighted values to each climatic model based on their rankings across all cities. For example, a model ranked first (Top 1) in a city is awarded 29 points, with a descending scale down to 1 point for the model ranked last (Top 29). These points are aggregated for each model across all cities to generate a cumulative score. We selected 17 models based on their score. This weighted value analysis provides a clear visual representation of each model’s overall performance, highlighting those that consistently ranked higher and were thus potentially more reliable for simulating heatwave characteristics.

### Temporal variation of heatwave index

To analyze the projected changes in heatwave characteristics over time, we assess the percentage difference in key heatwave indices between the historical baseline and future periods under both SSPs. The heatwave indices were computed annually throughout the historical baseline (1985–2014), near future (2025–2054), and far future (2065–2094) periods. For each year in the near future (2025–2054) and far future (2065–2094) periods, we calculated the percentage difference relative to the mean of the historical baseline period (1985–2014) using:

$$\:Percentage\:Change\:=\:\left(\frac{future\:-\:baseline}{baseline}\right)\:\times\:\:100$$

where ‘future’ represents the value for each year in the near future (2025–2054) or far future (2065–2094) periods, and ‘baseline’ represents the mean value from the historical period (1985–2014).

To summarize the signals across all cities within a given region, the heatwave indices and their percentage changes were averaged across the individual cities in each region. For each year, we then computed the multi-model median across all climate models to obtain a robust central estimate. This enables characterization of the projected temporal shifts in heatwave frequency, duration, intensity, and peak magnitude, specific to both daytime and nighttime extreme heat events. Examining both daytime and nighttime heat informs risks during vulnerable periods for human health and infrastructure operations^70,71^. Understanding how heatwave characteristics change under different SSPs is crucial for developing effective climate mitigation policies to reduce future heatwave risks.

### Composite heatwave index

We use a composite heatwave index (CHI)^29^ to integrate the multiple heatwave metrics and datasets into a singular representative index value for inter-comparison between cities and regions. The CHI accounts for the intensity, frequency, and duration characteristics across both daytime and nighttime using only the models that were fit for analysis^29^. The five previously computed heatwave indices (HWN, HWTD, HWLD, HWMT, HDT) are normalized on a scale of 0 to 1 for each city i and region across all CMIP6 models, time periods, and both daytime and nighttime extreme heat datasets. using min-max normalization.

For a given metric $\:j$ (with j = 1,2,3,4,5 for each heatwave metrics) for city $\:i$ and for a given dataset (daytime or nighttime), let the raw value be $\:{X}_{ij}$. We perform a min-max normalization across all 50 cities (for the same period and dataset):

$$\:{x}_{ij}=\frac{{X}_{ij}-{min}_{i}\left\{{X}_{ij}\right\}}{{max}_{i}\left\{{X}_{ij}\right\}-{min}_{i}\left\{{X}_{ij}\right\}}$$

This scales each metric to the interval [0,1] so that differences in units (days vs. $\:℃$) are eliminated. For each city $\:i$ and for each dataset separately, we aggregate the normalized metrics by taking their arithmetic mean. This, the composite index for city $\:i$ from, say, the daytime dataset is given by:

$$\:{\stackrel{-}{{x}_{i}}}^{\left(d\right)}=\frac{1}{5}\sum\:_{j=1}^{5}{x}_{ij}^{\left(d\right)}$$

Similarly, the nighttime composite index is:

$$\:{\stackrel{-}{{x}_{i}}}^{\left(n\right)}=\frac{1}{5}\sum\:_{j=1}^{5}{x}_{ij}^{\left(n\right)}$$

Since both daytime and nighttime extreme heat events are important for a comprehensive risk assessment, we combine the two datasets. We compute the overall CHI as:

$$\:CH{I}_{i}=\frac{{C}_{i}^{\left(d\right)}+{C}_{i}^{\left(n\right)}}{2}$$

After obtaining the combined CHI values for all cities, we perform a final min-max normalization to ensure that the CHI ranges from 0 to 1 across the study domain:

$$\:CH{I}_{i}^{final}=\frac{CH{I}_{i}-{min}_{i}\left\{CH{I}_{i}\right\}}{{max}_{i}\left\{CH{I}_{i}\right\}{-min}_{i}\left\{CH{I}_{i}\right\}}$$

Note that higher CHI values indicate greater cumulative heatwave hazard, with 1 representing the worst case. CHI enables quick visual differentiation of high-impact ‘hotspots’ versus lower-impact ‘cool spots’ across space, time, and climate uncertainty. The consolidated index retains a holistic perspective by equally weighing all duration, intensity, and frequency dimensions across daytime and nightime. The integrated city and regional CHI metrics are computed for historical, near future, and far future periods under both SSP-RCP scenarios (SSP2-4.5 and SSP5-8.5) for each climate model plus ensemble. This allows tracking projected progression of comprehensive heatwave risk across cities and regions over varying time horizons.

### Overlay of population dynamic and composite heatwave index

We identify urban zones facing the highest heat health risks. For this the 1 km gridded population projections^61^ under the five SSPs are overlaid onto the CHI maps for the corresponding future time periods. This integration analysis highlights areas where intense, frequent, and enduring heatwaves converge with dense and potentially vulnerable populations. We develop population heatmaps for the 2050s and 2090s under different SSP scenarios. By comparing these population heatmaps to the CHI, we identify hotspot areas experiencing both high heatwave intensity and increased future population. Apart from this, two distinct analyses were performed: (1) a direct risk assessment multiplying CHI with absolute population values to identify areas where high heat stress coincides with large population centers, and (2) a change-based risk assessment multiplying CHI with projected population changes to highlight areas where heat stress intersects with significant demographic shifts. Results were visualized through regional heatmaps organizing cities by geographic location (Northeast, Southeast, Midwest, Southwest, and West), enabling identification of “risk hotspots” where high heat hazard coincides with either large population centers or significant population growth. This dual analytical approach provides both absolute risk assessment (current population exposure) and dynamic risk evaluation (future population vulnerability) across different climate and socioeconomic scenarios. This can depict locations with both elevated CHI and high population totals, pinpointing ‘severe hotspots’ with the highest absolute numbers of vulnerable residents subject to extreme heat burdens in the coming future under different scenarios.

### Effect of air contaminants on increasing temperature and heatwave

The atmospheric composition data is derived from measurements collected by the TROPOspheric Monitoring Instrument (TROPOMI) aboard the Copernicus Sentinel-5P satellite^72^. Launched in 2017, Sentinel-5P provides global monitoring of key air quality trace gases and aerosols for advancing environmental and climate research^72^. Specifically, we utilize the daily Level 3 gridded absorbing aerosol index product quantifying ultra-violet aerosol loading across 0.01° × 0.01° grid cells from 2018. Additionally, the methane volume mixing ratio data is obtained from the same satellite platform on matching grids. Together, these contiguous spatial datasets offer a comprehensive resolution view into recent distribution patterns of critical particulate and greenhouse gas pollutants known to impact radiative transfer and regional heat dynamics.

### Coastal cities vs. Inland cities

Typically, inland cities have stronger heatwave effects compared to coastal cities^73^. The sea/ocean breeze has a strong cooling effect, especially during the nighttime for coastal cities, which also mitigates the increased temperature impacts during heatwaves. We examine the extent of such an effect from coast to coast across the US, particularly focusing on the West coast, South coast, and East-North coast. The comparative analysis is based on the selected 17 climate models. Heatwave characteristics during historical, near-term future and long-term future periods under both SSPs are examined for coastal and inland cities. The coastal cities are selected based on creating a 10 km buffer from the coast and considering them coastal cities which are intersected by these buffers.

## Results

### Comparison of reanalyzed data and climate models

Figure S1 presents a comparison of ERA5 data with ground-based observations, demonstrating the reliability of ERA5 for validation purposes. Out of the 50 selected urban areas, 49 exhibited an R^2^ greater than 0.95, while one had an R^2^ of 0.770, indicating a strong correlation between ERA5 and observed temperature data. Figure S2 provides a synopsis of the ranking of different downscaled CMIP6 climate model outputs as compared to ERA5 dataset. It is important to note that these results are based on downscaled CMIP6 model outputs, not direct model outputs, which allows for analysis at urban scales relevant to our study. The total maximum points a climate model could achieve in the weighted value analysis is 1450, based on a perfect score of ranking first across all 50 cities, with each first-place ranking worth 29 points. Despite the recent advances in climate models, inherent uncertainties and limitations in modeling complex climate systems prevent any model from achieving this theoretical maximum. Given that the maximum achievable points in the weighted value analysis are 1450, setting the threshold at half of this maximum, which is 725 points, serves as a reasonable criterion for selecting the most reliable climate models. According to this threshold, 17 climate models surpass the 725-point mark and are therefore selected for further consideration. These models demonstrate a higher level of accuracy and consistency in simulating heatwave indices across the 50 most populous US cities, making them valuable for detailed heatwave risk assessments and informing climate adaptation strategies. While Figure S2 presents the composite performance of climate models. A detailed analysis of individual heatwave indices (HWN, HWLD, HWTD, HDT, and HWMT) is also provided in the supplementary materials (Figs. S3–S7). This comprehensive breakdown reveals that although some models demonstrate consistent performance across all indices, others show varying capabilities in representing different heatwave characteristics. Hatched bars denote models selected for comprehensive analysis based on their robust performance in the composite index. For instance, certain models excel in capturing heatwave frequency (HWN, Fig. S3) and duration metrics (HWLD and HWTD, Figs. S4–S5), while others better represent temperature-based indices (HDT and HWMT, Figs. S6–S7). Despite these variations in individual metrics, the selected models (shown with hatched bars) generally maintain robust performance across multiple indices, supporting their selection for detailed analysis. Analysis of model characteristics reveals several key factors influencing performance in simulating urban heat extremes in Table S2. Models exceeding our 725-point threshold generally featured:

- Higher vertical resolution in the atmosphere (predominantly > 40 levels), allowing better representation of boundary layer processes crucial for urban temperature profiles. However, horizontal resolution alone did not determine performance, as evidenced by well-performing models with varying grid spacings (from ~ 80 km for EC-Earth3 to ~ 250 km for ACCESS-CM2).
- More sophisticated urban parameterizations, particularly those employing dedicated urban canopy models (e.g., Town Energy Balance (TEB) scheme in CNRM models, TERRA_URB in EC-Earth models) or multi-layer urban representations (e.g., CMCC-ESM2, NorESM2-MM). These schemes better capture urban-specific processes like anthropogenic heat release and building-atmosphere interactions compared to simple bulk schemes.
- Advanced convection and Planetary Boundary Layer (PBL) parameterizations, such as the PCMT prognostic scheme (CNRM models), Tiedtke-Bechtold scheme (EC-Earth models), and TKE-based PBL schemes. These parameterizations are crucial for representing the complex thermodynamic processes in urban atmospheric environments.

Conversely, models performing below our threshold typically featured simpler urban representations (bulk schemes or no explicit urban parameterization) and less sophisticated boundary layer physics, limiting their ability to capture urban heat characteristics accurately. Also, we found out that out of selected 17 models, 8 are AOGCMs and 9 are ESMs which suggest the model type is not a determining factor in performance for simulating urban heat extremes. The details on models used are shown in Table S2.

### Temporal variation of heatwave index

#### Frequency

**Fig. 2:**
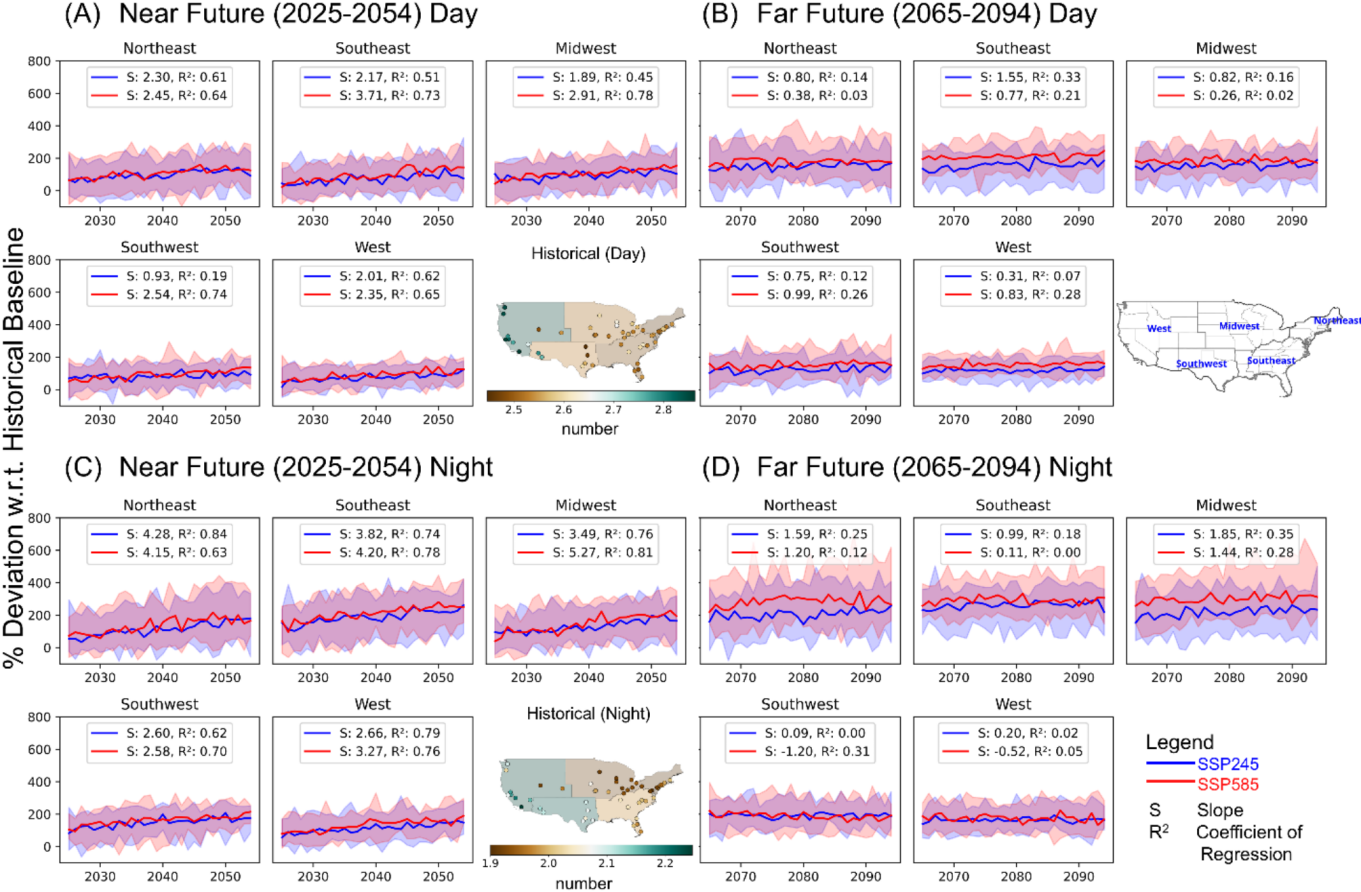
Projected percentage change in annual heatwave number (HWN) for daytime (**A**,**B**) and nighttime (**C**,**D**) in the near future (NF: 2025–2054) and far future (FF: 2065–2094) relative to the baseline period (1985–2014) across different regions by an ensemble median of the heatwave number shown by 17 selected climate models. Percentage changes are calculated as ((future − baseline)/baseline) × 100. Shaded areas represent the interquartile range (25th to 75th percentile) across the climate models, indicating the spread in model projections. The spatial map in panel (**A**) shows the daytime HWN during the historical baseline period, while the map in panel C shows the nighttime HWN during the baseline. Line graphs at panels (**A**) and (**C**) display the projections for the near future, while panels (**B**) and (**D**) show the far future projections. The blue line represents the SSP2-4.5 scenario, and the red line represents the SSP5-8.5 scenario, with corresponding slope and R^2^ values shown for each region to quantify the strength of temporal trends. Positive values indicate an increase in HWN while negative values indicate a decrease.

Figure 2 shows a projected change in heatwave numbers at different regions in NF and FF under different socioeconomic scenarios. These changes are calculated relative to each model’s own historical baseline period (1985–2014), rather than the ERA5 baseline, to ensure consistency and avoid potential biases from comparing model projections with reanalysis data. The spatial map in panel 2-A illustrates that in the baseline period (1985–2014), cities across the US experience an average of 2.5 to 2.9 daytime heatwaves annually. The West region and Southwest region cities are the most affected, experiencing a slightly higher number of daytime heatwaves. Panel 2-A shows the projected changes in daytime heatwave numbers for the near future, while panel 2-B displays the far future projections at daytime. Across all regions, substantial increases are projected relative to the baseline, with sharper rises under the SSP5-8.5 scenario (red line) compared to the SSP2-4.5 scenario (blue line).

Specifically, in the near future, daytime heatwave numbers are projected to increase by around 100% under SSP2-4.5 and 150% under SSP5-8.5 by the median ensemble. This means the average number of daytime heatwaves per year could reach 5 under SSP2-4.5 and 7 under SSP5-8.5. In the far future (Fig. 2B), the heatwave amplification continues, with all regions projected to see rises ranging from 200 to 300%. The Northeast, Southeast and Midwest are projected to experience maximum increase of around 250% (SSP2-4.5) to 300% (SSP5-8.5), leading to around 9.1–10.4 daytime heatwaves annually on average by the end of the century. The Southwest and West will also face continued rises, reaching up to an average of 200–250% more heatwaves than the baseline period.

The spatial map in panel 2-C shows that historically, an average of 1.9–2.3 nighttime heatwaves affected cities annually. The Southeast and West are most vulnerable in the baseline. Projections in panel 2-C and 2-D illustrate that all regions will experience major nighttime heatwaves increments in the near and far future like the daytime trends. The percentage rises range from 175 to 200% (SSP2-4.5) to 200–250% (SSP5-8.5) by the end-of-century. Unlike the daytime heatwaves, the Southwest and West show relatively steady projected growth for nighttime heatwaves in the future panels, while the Northeast, Southeast and Midwest illustrate continued sharp projected escalations. So, while historically less prone to night heatwaves, these regions appear to face greater future overnight heatwave risks.

#### Duration

The spatial map in Figure S8(A-D) indicates that during the historical period, Southwest and West region cities experience the greatest number of daytime heatwave days annually - around 11–15 days. Panel S8-A projects substantial rises in daytime heatwave duration across all regions in the near future, with increases of approximately 100% under SSP2-4.5 and 150% under SSP5-8.5. This suggests the average number of daytime heatwave days could reach 28 days per year under SSP2-4.5 and as many as 35 days under SSP5-8.5.

Sharper escalations are evident in the far future in Panel S8-B, showing approximately 250% rises under SSP2-4.5 and over 500% increases under SSP5-8.5 across most regions considering the ensemble median. Given high historical daytime heatwave durations, Southwest and West cities project relatively lower but still considerable percentage increases. Meanwhile, Northeast, Southeast and Midwest regions indicate particularly immense amplifications by the far future. If high emissions continue, annual daytime heatwave durations could approach 84 days on average across cities - nearly one fourth of the year. Under SSP2-4.5, 50 heatwave days per year are projected. Similarly, Panel S8-C shows historically 8–11 annual nighttime heatwave days affecting cities, most acutely in the Southwest and West. All areas are projected to face longer overnight heatwave durations. In the near future, nights could see around 22–27 heatwave affected nights under SSP2-4.5 and SSP5-8.5, respectively. More intense escalations are projected in the far future, potentially up to 33–40 nights annually under SSP2-4.5 and SSP5-8.5—over half the year facing night heatwaves. The Northeast, Southeast and Midwest are projected to experience acute overnight heatwave duration increases surpassing 500% above historical levels by some climate models.

Figure S9A–D in the supplementary section displays projected increases in the maximum annual heatwave duration (HWLD) for both daytime and nighttime heatwaves. Historically, the longest daytime heatwave duration affecting cities is around 5.5–7 days. Substantial rises in the daytime HWLD are projected for the near future under both SSP2-4.5 and SSP5-8.5, potentially reaching durations of 11–15 consecutive heatwave days and posing threats to infrastructure, health systems and more. Further intense amplifications are evident in the far future, when the SSP2-4.5 scenario projects maximum annual daytime heatwave durations approaching 14 days. Most concerning is the SSP5-8.5 trajectory, under which far future daytime heatwave events could span over a month (28 days) in length—a dangerous scenario. Similar patterns are projected for nighttime HWLD (Figure S9). While historically around 4–5 nights, maximum annual overnight heatwave durations could reach 7.5-9 nights by the near future and 13–20 nights under SSP2-4.5 and SSP5-8.5 respectively by the far future. These extreme prolonged night heatwaves would not allow relief overnight from intense heat for over half of the year by end-of-century (SSP5-8.5), with major implications for health and wellbeing.

#### Intensity

The spatial map in Figure S10-A shows that in the historical period, daytime mean temperatures during heatwaves range from 30 to 35 °C across the different US regions. Looking at the projected changes, we see a gradual rise in these heat wave intensities in both the near future and far future periods. In the near future, the ensemble median projects around 10–12% increases in daytime mean heatwave temperature (HWMT) under both SSPs. This suggests average daytime temperatures during heatwaves could reach 38–39 °C. However, a noticeable divergence emerges between the emissions scenarios in the far future (Fig. S10B). Under SSP2-4.5, daytime HWMT shows 15–20% rises from historical levels, potentially exceeding 40 °C on average regionally during heatwave events. The high emissions SSP5-8.5 trajectory drives even greater HWMT intensification of over 20%, with mean temperatures surpassing 42 °C in some regions. The exception is the West, which sees a relatively muted 10% HWMT rise given its already elevated historical levels shown in panel S10-A.

Turning to nighttime conditions, the spatial map at panel S10-C indicates historical overnight HWMT values of 17–21 °C. All regions are projected to experience considerable overnight heatwave intensification, even in the near future. Nighttime temperatures could warm up by 25–30% under SSP2-4.5 and SSP5-8.5 respectively, reaching around 24–25 °C—conditions disruptive for sleep and cooling needs^74–76^. More intense escalations manifest in the far future nighttime HWMT projections, with 30–40% increases over historical values (Figure S10-D). Under the high emissions SSP5-8.5, overnight temperatures exceeding 27 °C may become prevalent during heatwaves - extraordinarily warm for nighttime summer conditions historically. While lower under SSP2-4.5 at around 25 °C, overnight heatwave temperatures still show marked intensification even with emissions mitigation efforts.

Figure S11A–D in the supplementary section depicts projected changes in the hottest temperature experienced during annual heatwave events (HDT) for both daytime and nighttime conditions. Paralleling the patterns seen for mean heatwave temperatures, substantial increases in extreme HDTs are evident across future time periods and scenarios. Historically, the spatial map in panel A of Fig. S11 indicates daytime HDTs during annual heatwaves ranging from around 35–40 °C across US regions. Projections show rises of 10–15% in these extreme daytime temperatures by the near future under both emissions scenarios. This translates to the hottest heatwave days potentially reaching 42–45 °C regionally. More marked amplifications emerge for daytime HDT in the far future (Fig. S11B). Under SSP2-4.5, daytime HDTs could intensify 15–25% above historical levels, surpassing 45 °C in some areas during peak heatwave conditions. Yet the largest increases, upwards of 25–30%, materialize under the high emissions SSP5-8.5 trajectory. In this scenario, regions may face daytime HDTs exceeding a scorching 48 °C annually by the late century.

Similar patterns arise for nighttime HDT, albeit from cooler initial conditions historically in the 20–25 °C range (Fig. S11C). Near future projections indicate around 20–30% increases in overnight HDT under both scenarios. This raises the potential for peak overnight temperatures of 27–30 °C during heatwaves by mid-century. Far future nighttime HDT shows an even wider scenario divergence (Fig. S11D). SSP2-4.5 projects temperatures around 30 °C for the annual hottest heatwave night. However, SSP5-8.5 drives staggering potential increases over 45% from historical levels. Regions may face extreme overnight HDTs surpassing 32 °C annually—extraordinarily hot for nighttime conditions—if high emissions persist.

### Composite heatwave index

#### Historical (1985–2014)

Looking at the historical baseline period of 1985–2014, Fig. 3 shows the CHI computed across 50 cities and five regions by the selected 17 CMIP6 global climate models. Distinct spatial patterns emerge, with the highest index values concentrated in Southwestern and Western cities indicative of historical heatwave intensity hotspots.

**Fig. 3:**
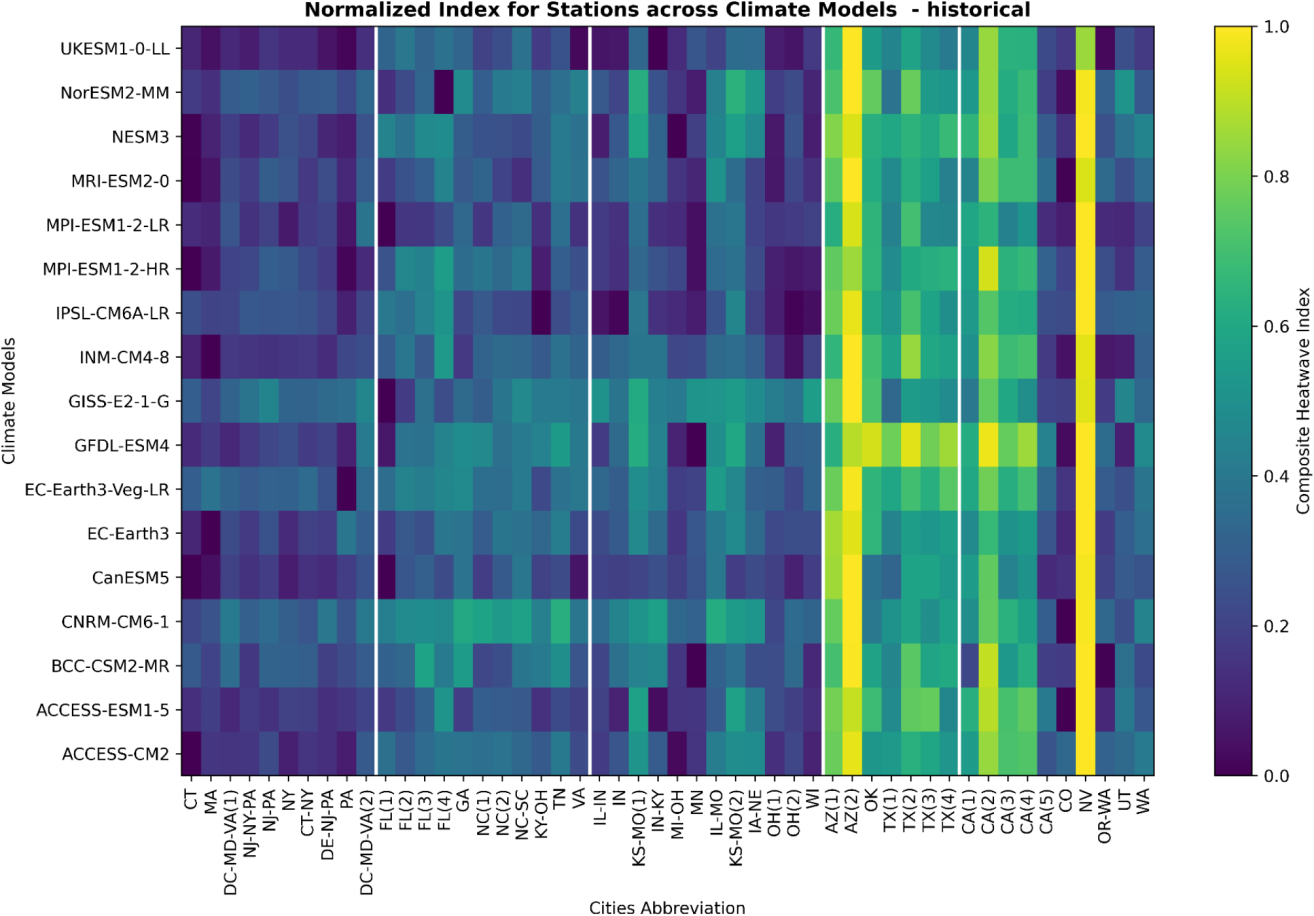
The composite heatwave index for different cities as simulated by the 17 selected CMIP6 global climate models during the 1985–2014 historical baseline period. Cities are grouped into the broader Northeast, Southeast, Midwest, Southwest, and West regions delineated by white lines. Refer to the Supplementary Data table for decoding the city ID to the corresponding names. Higher index values indicate locations facing more intense, frequent, and enduring historical heatwaves based on the model historical reanalysis.

The city of Las Vegas, NV (NV) stands out with a simulated historical CHI of 1.0 from 13 of the best ranked models, signaling it faced the most severe heatwaves from 1985 to 2014. Similarly, cities such as Yuma, AZ (AZ(2)) and Fresno, CA (CA(2)) metropolitan area also show heightened index values over 0.8 reflecting significant heatwave burdens during this period based on the model reanalysis and reconstruction.

#### Near future (2025–2054)

Examining the near future projections under the moderate emission scenario (Fig. S12), we see some notable changes from historical heatwave patterns. The Southwest region remains most vulnerable, with the highest composite heatwave index (CHI) values concentrated in cities like Yuma, Arizona (AZ2) and Las Vegas, Nevada (NV). These cities exhibited significant historical heatwave burdens and are projected to face further intensification in the coming decades. More surprisingly, the Miami metro area, represented by Broward, Miami-Dade and Palm Beach counties (FL1), emerges as the most heatwave-prone city in the near future projections (Fig. S12). These results highlight the potential for unprecedented severe future heatwaves across South Florida, beyond what historical trends would suggest.

Similar overall spatial patterns emerge under the higher emission scenario for the near future period (Fig. S13). The Southwest remains the most vulnerable region, with extreme CHI values concentrated around cities like Yuma, Arizona (AZ2) and Las Vegas, Nevada (NV). Again, these cities exhibited significant historical heatwave hazards and are projected to experience further intensification under high emissions in the coming decades. The Miami metro area (FL1) again stands out as the most heatwave-prone city in the SSP5-8.5 near future projections, despite inconsistent model projections. The potential for unprecedented severe future heatwaves affecting South Florida is clear, beyond what historical observations would suggest, especially under intense emissions.

#### Far future (2065–2094)

Looking further ahead to the far future period under the moderate scenario, Fig. S14 illustrates the Southwest remains the most vulnerable region to heatwaves. However, we see a shift towards heightened risks emerging across the Southeast and Midwest as well, as evident by more yellow/orange shading in those regions (Fig. S14). The Miami metro area (FL1) persists as the city with the highest projected composite heatwave index (CHI), affirming concerns about unprecedented future heat extremes in South Florida specifically. In addition, Southwest cities like Yuma, AZ (AZ2) & Pima, AZ (AZ1) and West city Las Vegas, NV (NV) remain hotspots given their substantial historical heatwave burdens.

**Fig. 4:**
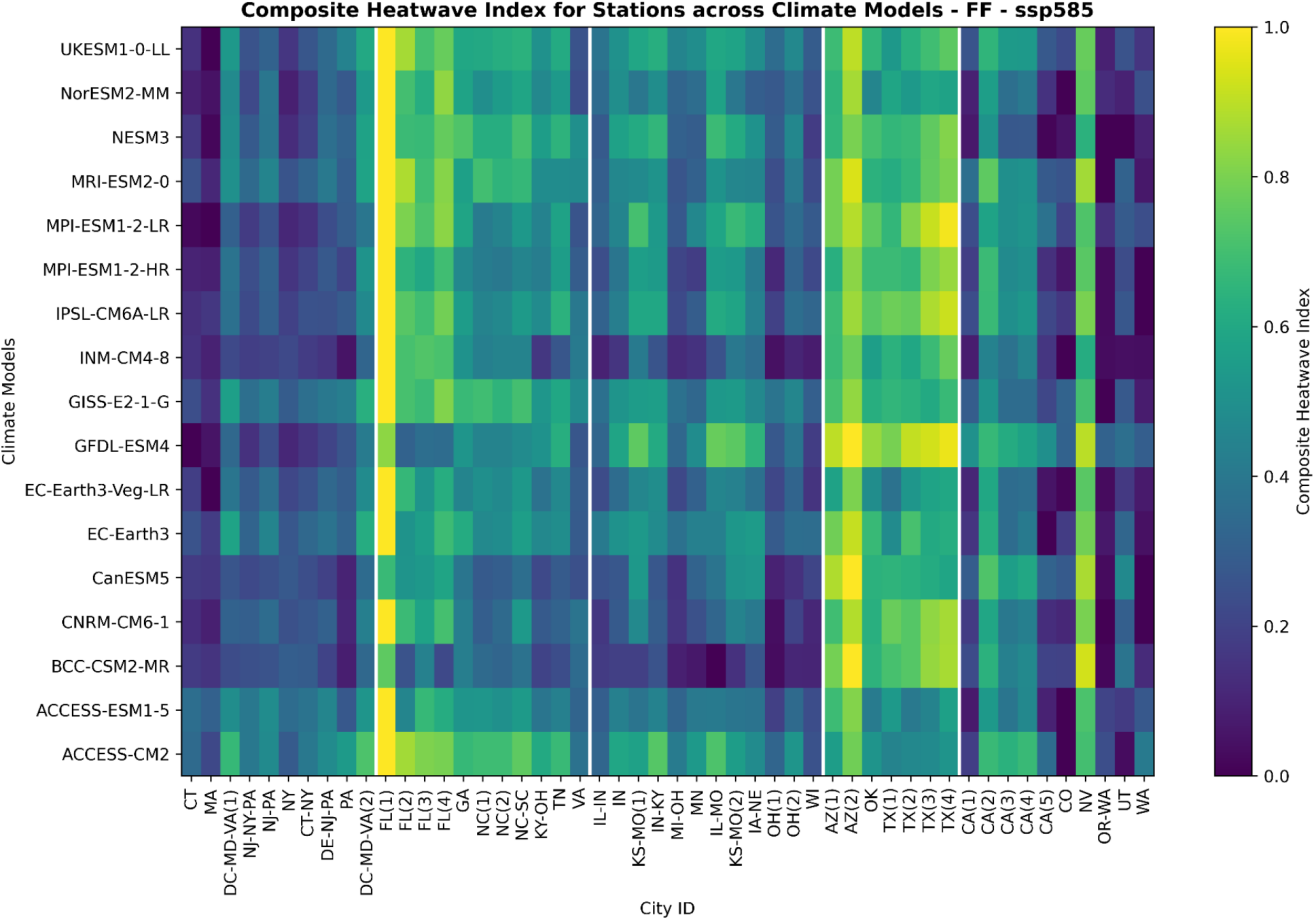
The composite heatwave index for different cities as simulated by the 17 selected CMIP6 global climate models during the 2065–2094 Far Future baseline period under SSP5-8.5. Cities are grouped into the broader Northeast, Southeast, Midwest, Southwest, and West regions delineated by white lines. Refer to the Supplementary Data table for decoding the city ID to the corresponding names. Higher index values indicate locations facing more intense, frequent, and enduring historical heatwaves based on the model SSP5-8.5 reanalysis.

Under the high emissions scenario in the far future period, the spatial patterns show more extensive heatwave risks across a larger geographic domain (Fig. 4). Intensifying CHI values depicted in yellow emerge across most regions, including the Southeast and Midwest. This indicates that uncontrolled emissions could expose many more cities across the US to unprecedented heat extremes later this century. The Southwest remains the hottest hotspot, with extreme risks projected across Arizona and Texas cities in particular. However, the hazarded areas are expanding outside of historical concentrations. The Miami metro region (FL1) again faces the most severe heatwaves in the projections. Cities across the Southwest like Yuma, AZ (AZ2), Las Vegas, NV (NV), and Tucson, AZ (AZ1) also remain at risk, given their substantial historical burdens.

### Overlay of population dynamic and composite heatwave index

#### Population in different SSPs

**Fig. 5:**
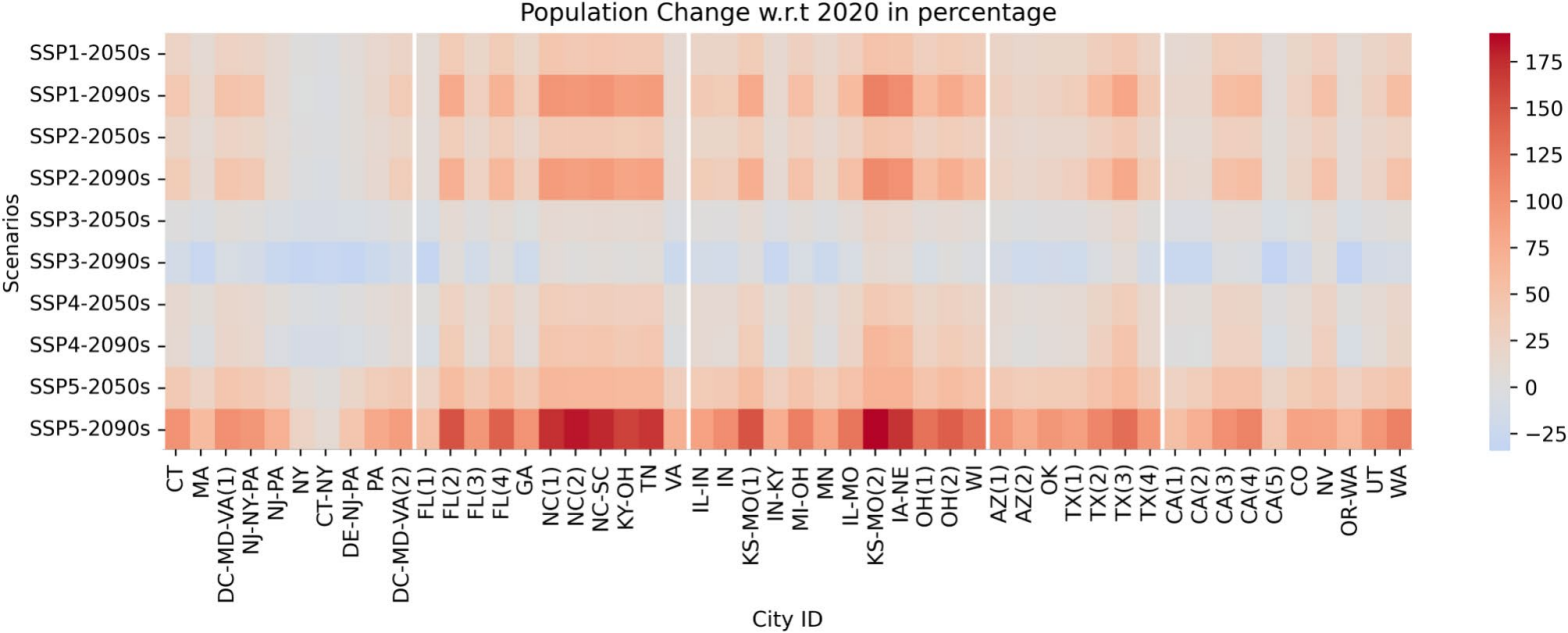
Heatmap of projected population changes by City ID. This figure illustrates the changes in population projected for the years 2050 and 2090 under different Shared Socioeconomic Pathways (SSPs), specifically SSP1, SSP2, SSP3, SSP4, and SSP5 scenarios. Each cell represents the urban area identified by the UID, showcasing the relative increase or decrease in population under each scenario. Color intensity correlates with the magnitude of population change, offering a visual representation of demographic shifts across various cities.

The projected population changes across study regions vary considerably depending on the SSP, reflecting different assumptions about fertility, mortality, migration, and economic development patterns as shown in Fig. 5. Under the sustainability focused SSP1, moderate population increases around—1.64–49.48% with an average 25% rise are projected by the near future period as education levels rise and fertility rates decline. Growth decelerates slightly in most places in the far future to − 4.29 to 115.78% as sustainability measures take effect. Regionally, we can see the higher increase of population in the Southeast in the Far Future whereas other regions show uniform rise. In the cities of Northeast (NY and CT) we can see a decrease in population in the SSP1 scenario.

The SSP2 “middle of the road” scenario, continuing current societal trends, sees slightly lower near-future growth of − 2.27 to 46.90% with an average 22% growth which tapers further to 42% average growth in different cities by the late century as the population gradually stabilizes. In contrast, the fragmented SSP3 narrative of regional rivalries and conflicts yields minimal to negative population changes by the 2050s (3% rise in average) that intensifies to more severe declines by the 2090s (− 11.61% in average) across certain regions. This scenario shows a range from − 9.45 to 19.18%, highlighting the possibility of population decline in some areas while others might see modest growth in Near Future whereas a range from − 34.18 to 13.82%, highlighting more pronounced declines across many regions, with a few areas still managing modest growth.

The inequality driven SSP4 future shows uneven demographic shifts, with modest 15.97% rises in some areas by mid-century alongside more substantial 40% increases elsewhere, reflecting concentrations of economic opportunities. Some areas in the Northeast even show a decrease of population by 4.37%. By 2090, more regions will begin exhibiting slight population declines (− 11.23%) as inequality exacerbates with some regions in Southeast and Midwest showing an increase up to 64.64%. However, the fossil-fueled development SSP5 trajectory leads to the highest population amplifications due to reduced mortality, high fertility, and migration drawn by economic growth. Increases exceed 40% by 2050 and approach or surpass 100% in certain regions by century’s end despite potential environmental degradation. The average change is around 42.72% at Near future, showing the highest increase among the scenarios, reflecting rapid economic growth and technological advancement. The average in Far Future change skyrockets to 104.09%, indicating a significant acceleration in population growth, underscoring the high-growth, high-consumption narrative of this scenario. We can see the growth up to 190% in some regions of Midwest and Southeast in the Far Future at SSP5.

#### Heatwave risk index

**Fig. 6:**
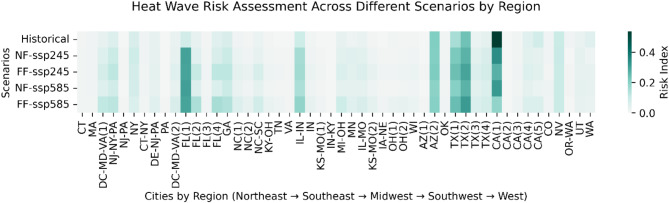
Heatmap of Absolute Heatwave Risk by City ID. This figure visualizes the spatial distribution of absolute heatwave risk across urban areas in the United States. The risk index is derived as the product of the composite heatwave index (normalized between 0 and 1) and the absolute population (also normalized), highlighting regions where extreme heat events and high population densities contribute to increased vulnerability. Darker shades indicate areas of higher risk.

The heatmap visualization in Fig. 6 represents the spatial and scenario-based variation in heatwave risk across different regions of the United States. The risk index, derived as the product of the composite heatwave index (normalized between 0 and 1) and the absolute population (also normalized), allows for the identification of regions where both extreme heat events and high population densities contribute significantly to overall risk. The Southwest (Arizona, Texas, and California), already facing high historical heatwave risk, continues to see significant increases in risk, particularly under scenarios of rapid urbanization such as SSP5 and SSP4. The Southeast, particularly Florida (FL(1)), and the Midwest (IL-IN region) stand out as areas where both population growth and heatwave intensity are projected to surge, making them new focal points of extreme heat exposure. The risk increase in Florida is particularly alarming, as cities that were historically at moderate risk could transition into high-risk zones due to combined demographic and climatic factors.

In contrast, areas with minimal population trends, such as parts of the Northeast and Upper Midwest, exhibit lower risk changes, primarily because stagnant population density lessens overall exposure. However, major metropolitan areas like New York City, Chicago, and Philadelphia still show moderate risk increases, reflecting the persistent urban heat island effect and the challenges of managing heat stress in densely populated environments.

In Fig. S15, the heatwave risk index is modified to account for population change with respect to 2020. This provides insights into how demographic shifts, in addition to climatic changes, influence future heatwave risk distributions. The stronger intensity of color in the South regions except in Northeast suggests that population growth amplifies heatwave risk in these areas. States such as Texas, Florida, and Arizona experienced pronounced increases in risk under future SSP scenarios, emphasizing the role of urban expansion and migration in shaping climate vulnerability. Comparing Fig. 6 and Fig. S15, key differences emerge in the spatial risk distribution. While the absolute population-based assessment emphasizes densely populated metropolitan areas, the population change-based analysis highlights rapidly growing regions where risk is projected to escalate significantly. This distinction is particularly evident in states like Florida and Texas, where rapid population growth compounds the impact of extreme heat. In contrast, regions such as the Northeast and Midwest experience relatively lower changes in risk due to either stable or declining population trends.

Some regions, particularly in the Southwest, demonstrate high risk levels in both analyses. These areas represent zones of compound vulnerability where large current populations coincide with projected demographic growth in areas of high heat stress. This dual-risk profile suggests these regions require comprehensive adaptation strategies addressing both immediate and future challenges. The Southeast presents an interesting case where moderate absolute risk levels contrast with high population-change-based risk. This pattern indicates potential future hotspots where current heat management strategies may need significant enhancement to address growing population exposure. The Northeast and Midwest generally show lower risk values in both analyses, suggesting these regions might have greater adaptive capacity. However, this should not lead to complacency, as moderate risk levels still indicate significant vulnerability.

## Discussion

### Effects of air contaminants on increasing temperature

Our analysis reveals heightened atmospheric aerosol and methane concentrations coinciding with regions facing the most severe heatwaves, hinting at an interplay between air quality and extreme heat amplification. Southwest and western cities exhibit both high composite heatwave index values (Figs. 3 and 4, S12–S14) and elevated aerosol optical depth and methane column densities (Fig. S16) based on historical satellite remote sensing records. These spatial correlations indicate a potential interplay between urban air pollution and extreme heat events, though further investigation is required to establish definitive causal mechanisms. The analysis of aerosol and methane concentrations spans the period 2018/07/10–2023/12/31 for aerosol concentration and 2019/02/08-2023/12/31 for methane concentration based on its availability, rather than being limited to heatwave days, allowing for a broader assessment of long-term patterns.

However, substantial uncertainties remain regarding the direct linkage mechanisms and causal pathways. Potential explanatory factors behind the apparent correlations include: (1) heatwave-conducive meteorological conditions also favoring atmospheric stagnation that traps pollutants^77,78^, (2) higher solar radiation driving photochemical reactions amplifying aerosols^79,80^, (3) heat-induced emissions from built infrastructures and disturbed biogeochemical cycles^79,81^, and (4) positive feedback where the air pollutants’ radiative effects further exacerbate urban heat^81,82^. Disentangling these interconnected processes requires targeted simulation studies together with lagged and isopleth spatiotemporal correlation analysis. If robust mechanistic attribution can quantify contributory effects of regional air pollution on heat extremes, then cross-sectoral mitigation approaches tackling both climate and public health objectives may provide optimally holistic resilience solutions.

### Coastal vs. Inland cities

**Fig. 7:**
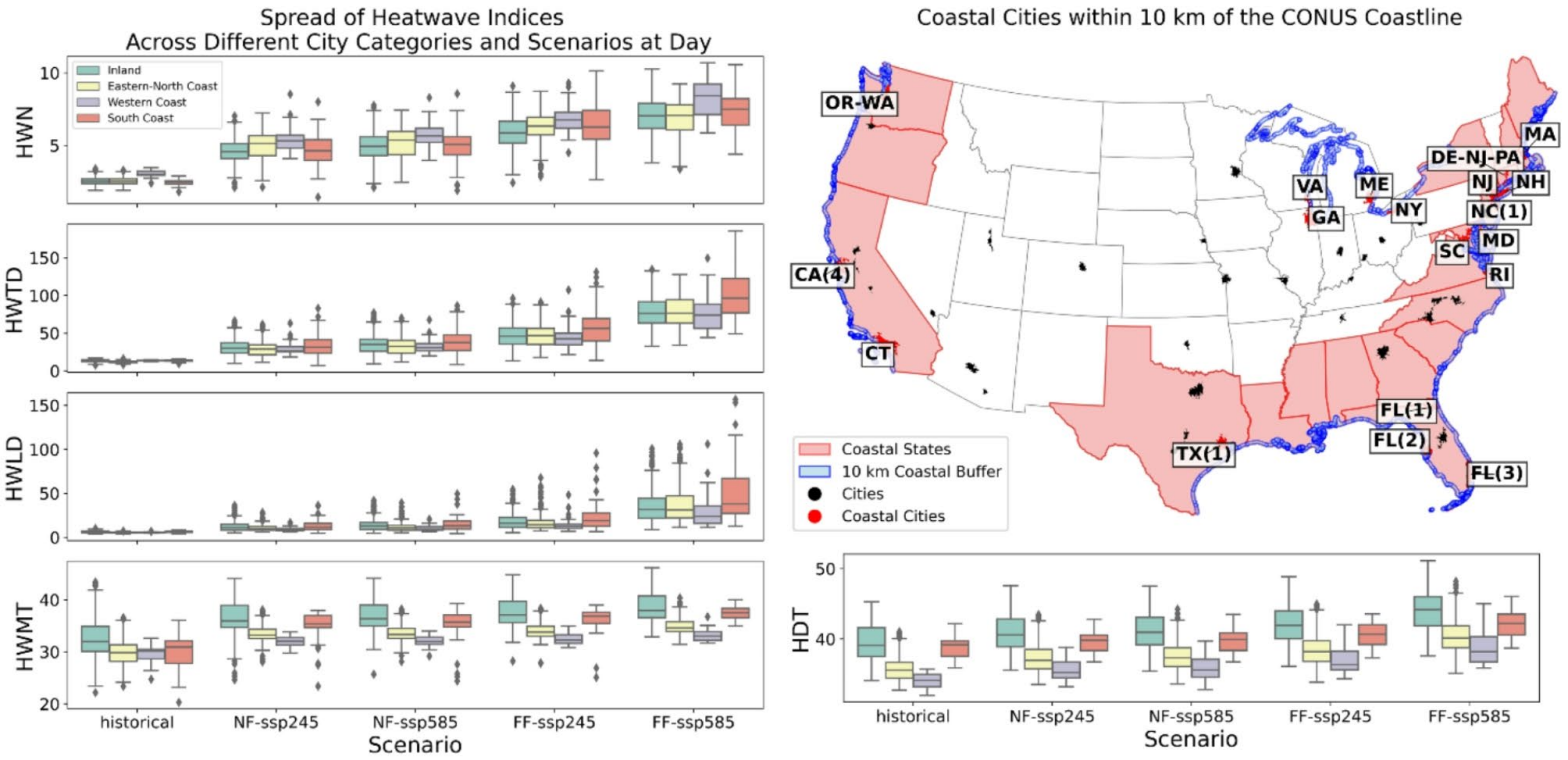
Spatial distribution of coastal cities across the Western Coast, South Coast, and East-North Coast regions, identified by creating a 10-km buffer from the coastline. The cities intersected by the buffer (represented by blue line) are classified as coastal cities and categorized into the respective coastal regions. Box plots illustrate the heatwave characteristics, including frequency (HWN), duration (HWTD and HWLD), and intensity (HWMT and HDT), across different scenarios and regions during the day. The regions comprise the Western Coast, South Coast, East-North Coast, and inland areas. In the box plots, the center line represents the median, the box shows the interquartile range (25th to 75th percentiles), and the whiskers extend to 1.5 times the interquartile range beyond the box edges. Points beyond the whiskers represent statistical outliers.

The box plots in Fig. 7 provide a comparison across different scenarios and regions. In general, the coastal areas at different positions exhibited different heatwave characteristics compared to the inland areas. In terms of frequency, coastal areas tend to experience a higher number of heatwaves compared to inland regions, a pattern consistent across historical and projected scenarios. Specifically, the western coastal cities exhibit the highest number of heatwaves in every historical and projected scenario. The ranking then proceeds towards the South Coast, East-North Coast, and inland cities. The number of heatwaves is increasing in all these cities under future projections, with the western coast cities exhibiting the most substantial increase.

In terms of duration and intensity, during the historical timeframe, the average median duration of heatwaves (measured as both the total number of heatwave days and the longest heatwave duration) is relatively similar across all cities and regions. However, in future projections, disparities emerge, with the South Coast (especially in Florida) exhibiting an aggressive increase in heatwave duration characteristics. Consistent with the frequency pattern, the coastal cities generally experience longer heatwave durations compared to inland areas, as evident in Fig. 7. The average median intensity across all cities and regions reveals maximum impact in inland areas. Regarding temperature intensity, the coastal cities appear substantially cooler, with the western coast being the coolest during the day. The inland cities experience higher temperatures than the coastal cities. However, from the historical perspective, the temperature increase trend is more pronounced in coastal cities, as demonstrated in Fig. 7.

The analysis revealed notable findings for nighttime heatwaves as well. Surprisingly, the South Coast areas exhibited larger values across all heatwave characteristics, including frequency, duration, and even intensity. Both historical data and future projections consistently showed higher nighttime heatwave exposure in these regions compared to other areas. The higher frequency and duration of heatwaves in the Coast regions can be attributed to the high specific heat capacity of water bodies, which tend to moderate temperature changes near the coast. The inland cities, on the other hand, demonstrated lower vulnerability in terms of frequency and duration, but exhibited higher intensity levels akin to the South Coast, as evident in Fig. S17 in the supplement section. This can be explained by the natural temperature patterns, where the inland regions historically experience higher temperatures compared to the coastal areas. The findings suggest that the coastal regions, particularly the South Coast, are more susceptible to the impacts of marine heatwaves and sea dynamics, which can influence the frequency and duration of nighttime heatwaves. Further quantification is necessary to determine the extent to which these factors contribute to the observed patterns in heatwave characteristics across the different regions.

The regional analysis in the result section provided insights into the variations in heatwave characteristics across different regions. However, it is crucial to acknowledge the distinct differences between coastal and inland cities that emerged from the analysis. The disparity of coastal and inner cities was somehow lost in the regional study. Our analysis reveals distinct regional patterns across different heatwave characteristics: western coastal cities experience the highest frequency of heatwaves, the South Coast region shows the most pronounced duration of heatwave events, while inland cities exhibit the highest temperature intensities during heatwaves. This nuanced pattern highlights the importance of considering multiple heatwave metrics when assessing regional vulnerability. These findings underscore the importance of examining the coastal-inland divide in addition to regional variations. The unique geographical and climatic factors associated with coastal and inland areas can significantly influence heatwave patterns and impacts.

### Urban population exposure to heatwaves

SSP1 (Sustainability) and SSP2 (Middle of the Road) represent scenarios with moderate to high sustainability efforts and technological advancement, which can be somewhat aligned with the climate scenario SSP2-4.5, reflecting intermediate greenhouse gas emissions and moderate global warming. Figure 5 reveals that higher population changes are observed in the Southeast and Midwest regions, which are comparatively smaller CHI zones. Therefore, when planning heat resilience strategies, it is crucial not to focus on areas with high heat hazards, but also distribution of population exposed. In cities experiencing moderate population increases under SSP1 and SSP2, if urban planning incorporates sustainability measures, the rise in CHI under SSP2-4.5 might be mitigated through green infrastructure, improved urban layouts, and heatwave response strategies. However, without sufficient adaptation measures, even moderate population growth could exacerbate heatwave conditions and their impacts on urban populations. Therefore, proactive urban planning that integrates climate change adaptation and mitigation strategies is essential to ensure urban resilience and safeguard public health under various climate and socioeconomic scenarios.

SSP3 (Regional Rivalry) reflects a world with fragmented policies and limited global cooperation. This could indirectly lead to scenarios where mitigation efforts are not fully effective, somewhat akin to a neglected version of SSP2-4.5 - despite not reaching the extreme conditions of SSP5-8.5, the lack of a coordinated response could worsen the impacts of heatwaves. In cities under SSP3, the minimal or negative population changes could reduce pressure on resources but might also reflect a lack of capacity to implement effective heatwave mitigation strategies; despite potentially lower population growth, these cities might still face increasing CHI under SSP2-4.5 due to neglected infrastructure and poor urban planning, exacerbating the adverse effects of heatwaves on their populations.

SSP5 (Fossil-fueled Development) reflects high economic growth fueled by fossil energy use, aligning closely with the high emission scenario SSP5-8.5, where heatwaves become more intense and frequent. The population is increasing everywhere under SSP5, and the composite heatwave index is observed to be approaching 1, indicating severe heatwave conditions across most regions. The temporal plots in Fig. 2, S8-11 also illustrate the devastating impact of the SSP5-8.5 scenario on various heatwave characteristics, highlighting its dangerous implications. Cities under SSP5 experiencing significant population growth are likely to face severe increases in CHI under SSP5-8.5 due to high emissions and intensified urban heat island effects; this scenario would necessitate aggressive heatwave mitigation strategies, particularly in rapidly expanding urban areas. The combination of widespread population growth and extreme heatwave conditions under SSP5-8.5 presents a formidable challenge, requiring urgent and comprehensive measures to safeguard public health and ensure urban resilience.

The analysis of heatwave risk across different SSP scenarios reveals complex interactions between population dynamics and climate change impacts. The Southwest region, particularly Arizona, Texas, and California, demonstrates persistently high risk levels across all scenarios, with risk indices approaching 1 under SSP5-8.5, indicating severe heatwave conditions. This region’s vulnerability is compounded by significant population growth projected under SSP5, intensifying the urban heat island effect and necessitating comprehensive adaptation strategies. The Southeast, particularly Florida, emerges as a critical focal point where moderate baseline risk transforms into high-risk projections due to the combined effects of population growth and increasing heatwave intensity. Under SSP1 and SSP2 scenarios, aligned with SSP2-4.5 climate projections, these regions show moderate increases in composite heatwave index (CHI), suggesting potential for mitigation through sustainable urban planning and green infrastructure implementation. However, the effectiveness of these measures heavily depends on proactive policy implementation and regional cooperation.

### Previous studies

The findings of this study align with previous research conducted by the US Global Change Research Program, which analyzed changing heatwave trends and characteristics from 1961 to 2021^58,83^. Their analysis also showed an increasing trend in the average number of heatwaves and total heatwave duration in the top 50 metropolitan areas by decade, with the western, southwestern, and Miami regions being the most affected^58,83^. The definition of a heatwave in their study is little different from that we adopted in this study, considering an event when temperatures exceeded the 85th percentile for two or more consecutive days but the spatial map showed the same distribution. Our historical spatial representation of heatwave characteristics is also consistent with the study, which utilized NOAA data over a historical time frame^57^. Notably, the Miami area has recently garnered attention for breaking several previous records, both for land and marine heatwaves, including a massive 37-day heatwave, corroborating our projections for the near future period^84,85^. Furthermore, the rapid increase in heat-related mortality, which has risen by 95% from 2010 to 2022, highlights the urgency of the situation and aligns with our findings of increased severity in the CHI for the Miami region, particularly in the near future and far future periods under both SSPs^86^. However, previous studies have not projected these indices, which are crucial for urban planners, policymakers, and the nation.

## Conclusions

This study provides a comprehensive assessment of heatwave impacts across highly populated US cities, projecting future scenarios and exploring the relationship between heatwaves and their associated health and environmental consequences. By analyzing spatial and temporal patterns of heatwave intensity, frequency, and duration, we identify distinct regional variations and contrasting impacts between coastal and inland urban areas. The regional analysis revealed notable disparities, with the Southwest region consistently emerging as the most vulnerable hotspot for intense, frequent, and prolonged heatwaves across historical and projected scenarios. However, risks are anticipated to escalate across the Southeast and Midwest as well, particularly under higher emissions trajectories in the far future. Recognizing these regional variations is crucial for tailoring heatwave mitigation and adaptation strategies to address the unique challenges faced by different geographic areas. Even under moderate emissions scenarios, all regions are expected to face substantial amplification in heatwave characteristics by the mid and late century. The high emissions trajectories aligned with fossil-fueled development portend unprecedented heat extremes becoming widespread, with severe implications for public health, infrastructure resilience, and urban livability.

Overlay analysis with projected population distributions revealed potential hotspots where dense populations intersect with intense, frequent heatwaves. This confluence underscores the urgency of implementing comprehensive adaptation and mitigation strategies, tailored to the specific needs of vulnerable communities and regions. Our findings emphasize the necessity of examining both regional variations and the coastal-inland divide when assessing heatwave risks and developing targeted interventions. The unique geographical and climatic factors influencing these disparities must be accounted for to enhance urban resilience effectively. Ultimately, this research highlights the pressing need for proactive urban planning that integrates climate change adaptation and mitigation strategies. By considering both climate hazards and socioeconomic vulnerabilities, policymakers and urban planners can allocate resources optimally, prioritize interventions, and safeguard public health and well-being in the face of escalating heatwave challenges under various climate and population scenarios.

## Electronic supplementary material

Below is the link to the electronic supplementary material.

Supplementary Material 1

## Data Availability

The shapefile of 247 large US cities was obtained from Chen, B. et al. (2022), available at 10.1038/s41467-022-32258-4. The 1 km × 1 km resolution population data, available in TIFF format, was downloaded from 10.1038/s41597-022-01675-x. This data was used to filter the top 50 most populous cities in the US. The CMIP6 datasets were accessed from the NASA GDDP-CMIP6 catalog (https://developers.google.com/earth-engine/datasets/catalog/NASA_GDDP-CMIP6), and the ERA5 data were obtained from the ECMWF_ERA5_LAND_DAILY_AGGR catalog (https://developers.google.com/earth-engine/datasets/catalog/ECMWF_ERA5_LAND_DAILY_AGGR), both of which were downloaded using Google Earth Engine. The data used for this analysis, after downloading from Google Earth Engine, are available on Zenodo: Bhattarai, S. (2024). CMIP6 Tmax and Tmin Data for 50 Most Populous Cities in USA (Version 1.0.0) [Data set]. Zenodo. 10.5281/zenodo.10998542. All or part of the code will be made available upon request to the corresponding author. We used freely available codes such as Geopandas, Numpy, Pandas, Matplotib, and Seaborn for data analysis and graph production.
