# Supplementary material for: Understanding spatiotemporal variation of heatwave projections across US cities
Source: Sci Rep. 2025 Mar 27;15:10643. doi: 10.1038/s41598-025-95097-5 (PMC11950315; doi:10.1038/s41598-025-95097-5)
Supplement: Supplementary file 1 — Supplementary Material 1 [file 41598_2025_95097_MOESM1_ESM.docx]

**Spatiotemporal characterization of heatwave projections across US cities**

**Saurav Bhattarai^1,^ *, Laxman Bokati^2^, Sanjib Sharma^3^, Rocky Talchabhadel^1^**

^1^Department of Civil and Environmental Engineering, Jackson State University, Jackson, MS, USA

^2^School of Sustainable Engineering and the Built Environment, Arizona State University, Tempe, AZ, USA

^3^Department of Civil and Environmental Engineering, Howard University, Washington, DC, USA

Corresponding Author: Saurav Bhattarai ([saurav.bhattarai@students.jsums.edu](mailto:saurav.bhattarai@students.jsums.edu))

**Contents of this file**

Table S1-S2

Figures S1 to S18

**Supplementary**

**Table S1.** City ID alongside the urban clusters within each identified city region.

| **City ID** | **City Name** |
| --- | --- |
| AZ(1) | Pima |
| AZ(2) | Maricopa, Pinal |
| CA(1) | Los Angeles, Orange, Riverside, San Bernardino, San Diego, Ventura |
| CA(2) | Fresno, Madera, Tulare |
| CA(3) | Merced, San Joaquin, Stanislaus |
| CA(4) | El Dorado, Placer, Sacramento, Yolo |
| CA(5) | Alameda, Contra Costa, San Francisco, San Mateo, Santa Clara |
| CO | Adams, Arapahoe, Boulder, Broomfield, Denver, Douglas, Jefferson |
| CT | Fairfield, Hartford, Litchfield, Middlesex, New Haven, Tolland |
| FL(1) | Broward, Martin, Miami-Dade, Palm Beach |
| FL(2) | Lake, Orange, Osceola, Polk, Seminole, Volusia |
| FL(3) | Clay, Duval, Saint Johns |
| FL(4) | Hernando, Hillsborough, Pasco, Pinellas |
| GA | Bartow, Cherokee, Clayton, Cobb, DeKalb, Douglas, Fayette, Forsyth, Fulton, Gwinnett, Hall, Henry, Newton, Paulding, Rockdale, Walton |
| IL-IN | Cook, Dupage, Grundy, Kane, Kendall, Lake Michigan, Lake, McHenry, Will, Lake |
| IN | Boone, Hamilton, Hancock, Hendricks, Johnson, Marion, Morgan |
| KS-MO(1) | Johnson, Leavenworth, Miami, Wyandotte, Cass, Clay, Jackson, Platte |
| IN-KY | Clark, Floyd, Bullitt, Jefferson, Oldham |
| MA | Bristol, Essex, Middlesex, Norfolk, Plymouth, Suffolk |
| DC-MD-VA(1) | District of Columbia, Anne Arundel, Baltimore, Howard, Montgomery, Prince George's, Fairfax |
| MI-OH | Lake St. Clair, Macomb, Monroe, Oakland, Saint Clair, Washtenaw, Wayne, Lake Erie |
| MN | Anoka, Carver, Dakota, Hennepin, Ramsey, Scott, Sherburne, Washington, Wright |
| IL-MO | Madison, Saint Clair, Jefferson, Saint Charles, Saint Louis, Warren |
| KS-MO(2) | Johnson, Wyandotte, Cass, Clay, Jackson, Platte |
| NC(1) | Chatham, Durham, Franklin, Granville, Harnett, Johnston, Orange, Wake, Wilson |
| NC(2) | Alamance, Davidson, Davie, Forsyth, Guilford, Orange, Randolph |
| NC-SC | Cabarrus, Catawba, Cleveland, Gaston, Iredell, Lincoln, Mecklenburg, Rowan, Union, Lancaster, York |
| IA-NE | Pottawattamie, Douglas, Sarpy, Washington |
| NJ-NY-PA | Bergen, Burlington, Essex, Hudson, Hunterdon, Mercer, Middlesex, Monmouth, Morris, Ocean, Passaic, Somerset, Sussex, Union, Rockland, Bucks |
| NJ-PA | Burlington, Camden, Gloucester, Bucks, Philadelphia |
| NV | Clark |
| NY | Kings, Nassau, Queens, Suffolk |
| CT-NY | Fairfield, Bronx, New York, Westchester |
| OH(1) | Cuyahoga, Geauga, Lake Erie, Lake, Lorain, Medina, Portage, Summit |
| OH(2) | Delaware, Fairfield, Franklin, Licking, Pickaway, Union |
| KY-OH | Campbell, Kenton, Butler, Clermont, Hamilton, Warren |
| OK | Canadian, Cleveland, Logan, McClain, Oklahoma |
| OR-WA | Clackamas, Multnomah, Washington, Clark |
| DE-NJ-PA | New Castle, Burlington, Camden, Mercer, Berks, Bucks, Chester, Delaware, Montgomery, Philadelphia |
| PA | Allegheny, Beaver, Butler, Washington, Westmoreland |
| TN | Davidson, Rutherford, Sumner, Williamson, Wilson |
| TX(1) | Brazoria, Fort Bend, Galveston, Harris, Liberty, Montgomery, Waller |
| TX(2) | Collin, Dallas, Denton, Ellis, Johnson, Kaufman, Rockwall, Tarrant, Wise |
| TX(3) | Hays, Travis, Williamson |
| TX(4) | Bexar, Comal, Guadalupe |
| UT | Davis, Salt Lake, Utah, Weber |
| VA | Chesapeake, Norfolk, Portsmouth, Suffolk, Virginia Beach |
| DC-MD-VA(2) | District of Columbia, Montgomery, Alexandria, Arlington, Fairfax City, Fairfax, Falls Church, Loudoun, Manassas Park, Manassas, Prince William |
| WA | King, Pierce, Snohomish |
| WI | Kenosha, Lake Michigan, Milwaukee, Ozaukee, Racine, Washington, Waukesha |


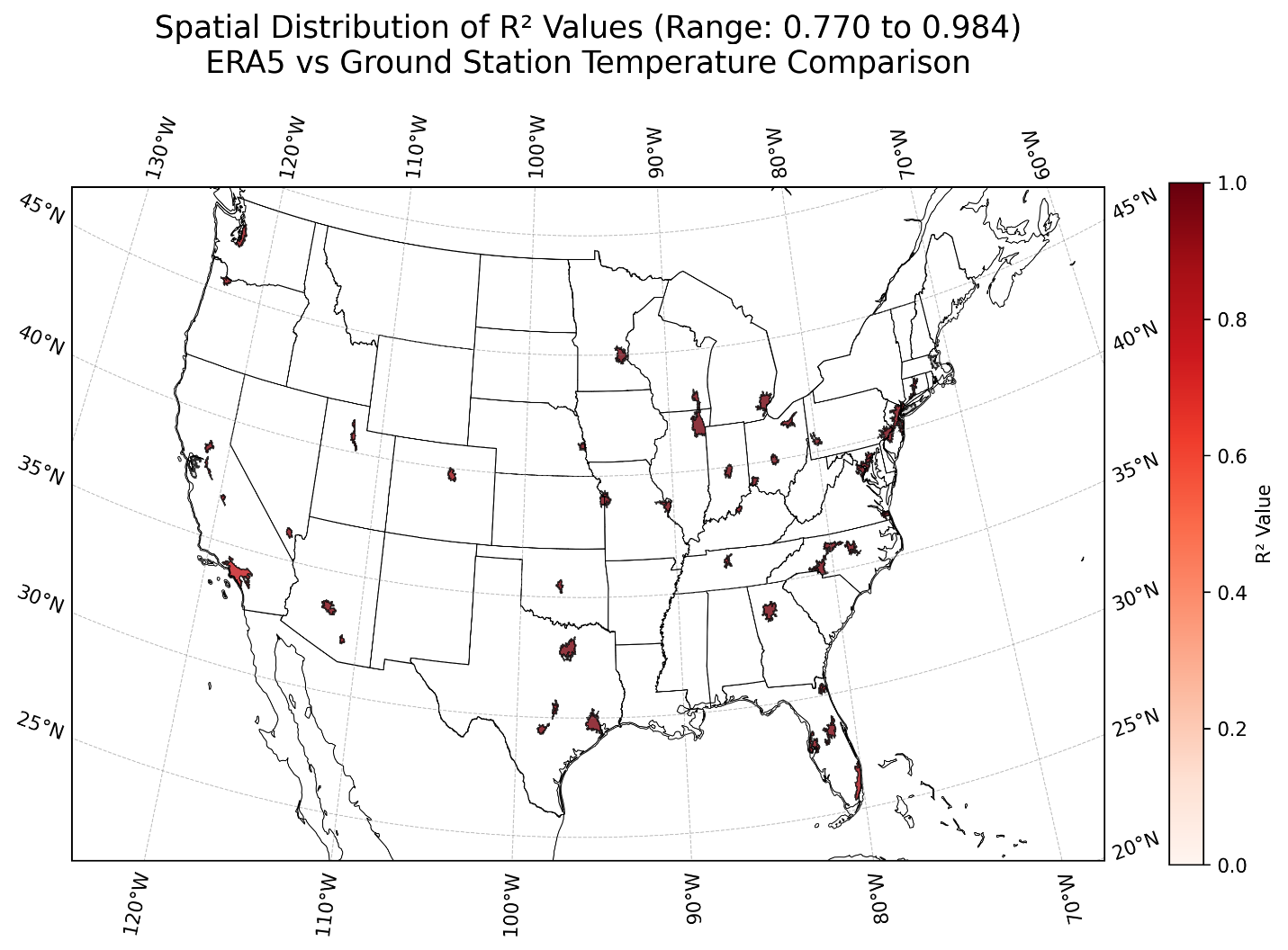


**Figure S1.** Spatial distribution of R² values comparing ERA5 daily average 2m air temperature with ground-based observations across 50 urban areas in the contiguous United States. The map illustrates the reliability of ERA5 in capturing local temperature variations, with most urban areas showing strong correlations (R² > 0.95). State boundaries are overlaid for reference. The analysis was performed using the Meteostat Python library, and R² values were computed to validate ERA5 as a dataset for climate model evaluation.

Table S2: Comprehensive Characteristics of All CMIP6 Models Used in This Study. Models are listed alphabetically, with asterisks (*) indicating models that exceeded our 725-point threshold for accurate representation of urban heat extremes.

| **Model** | **Model Type** | **Institution** | **Atmosphere Resolution** | **Ocean Resolution** | **Vertical Levels (Atm/Ocean)** | **Land Surface Model** | **Urban Scheme** | **Convection Scheme** | **Radiation Scheme** | **PBL Scheme** |
| --- | --- | --- | --- | --- | --- | --- | --- | --- | --- | --- |
| ACCESS-CM2* | AOGCM | CSIRO-BOM, Australia | 1.875° × 1.25° | 1° × 1° | 85/50 | CABLE2.5 | Bulk urban scheme | Mass flux with deep/shallow convection | SOCRATES | MO Large-eddy |
| ACCESS-ESM1-5* | ESM | CSIRO, Australia | 1.875° × 1.25° | 1° × 1° | 38/50 | CABLE2.4 with CASA-CNP | Bulk urban scheme | Modified Gregory-Rowntree | SOCRATES | MO Large-eddy |
| AWI-CM-1-1-MR | AOGCM | AWI, Germany | T127 (~100km) | 0.5° × 0.5° | 95/46 | JSBACH3.2 | No explicit scheme | Tiedtke-Nordeng | RRTM-G | TKE-based |
| BCC-CSM2-MR* | AOGCM | BCC, China | T106 (~100km) | 1° × 1° | 46/40 | BCC_AVIM2 | Single-layer urban | Mass flux with updated closure | BCC-RAD | UW-PBL |
| BCC-ESM1 | ESM | BCC, China | T42 (~280km) | 1° × 1° | 26/40 | BCC_AVIM2 | Simple bulk | Mass flux scheme | BCC-RAD | UW-PBL |
| CAMS-CSM1-0 | AOGCM | CAMS, China | 1° × 1° | 1° × 1° | 31/50 | CLM4.0 | No explicit scheme | Zhang-McFarlane | RRTM-G | HB |
| CAS-ESM2-0 | ESM | CAS, China | T128 (~100km) | 1° × 1° | 35/30 | CoLM | Simple bulk | Mass flux scheme | RRTM-G | YSU |
| CESM2 | ESM | NCAR, USA | 0.9° × 1.25° | 1° × 1° | 32/60 | CLM5 | Multi-layer urban | Zhang-McFarlane | RRTM-G | HB |
| CMCC-CM2-SR5 | AOGCM | CMCC, Italy | 1° × 1° | 1° × 1° | 30/50 | CLM4.5 | Single-layer urban | Modified Tiedtke | RRTM-G | YSU |
| CMCC-ESM2* | ESM | CMCC, Italy | 1° × 1° | 1° × 1° | 46/50 | CLM4.5 | Multi-layer urban | Mass flux with CAPE | RRTM-G | YSU |
| CNRM-CM6-1* | AOGCM | CNRM-CERFACS, France | T127 (~150km) | 1° × 1° | 91/75 | SURFEX v8 | TEB scheme | PCMT prognostic | RRTM-G | CBR |
| CNRM-ESM2-1* | ESM | CNRM-CERFACS, France | T127 (~150km) | 1° × 1° | 91/75 | SURFEX v8 | TEB scheme | PCMT prognostic | RRTM-G | CBR |
| EC-Earth3* | AOGCM | EC-Earth Consortium | T255 (~80km) | 1° × 1° | 91/75 | HTESSEL | TERRA_URB | Tiedtke-Bechtold | RRTM-G | K-diffusion |
| EC-Earth3-Veg* | ESM | EC-Earth Consortium | T255 (~80km) | 1° × 1° | 91/75 | HTESSEL | TERRA_URB | Tiedtke-Bechtold | RRTM-G | K-diffusion |
| FGOALS-f3-L | AOGCM | CAS, China | 1° × 1° | 1° × 1° | 32/30 | CLM4.0 | Simple bulk | Zhang-McFarlane | RRTM-G | HB |
| FGOALS-g3* | ESM | CAS, China | 2° × 2° | 1° × 1° | 26/30 | CLM4.0 | Single-layer urban | Mass flux scheme | RRTM-G | HB |
| FIO-ESM-2-0 | ESM | FIO, China | T42 (~280km) | 1° × 1° | 26/40 | CLM4.0 | No explicit scheme | Zhang-McFarlane | RRTM-G | HB |
| GFDL-CM4 | AOGCM | NOAA-GFDL, USA | 1° × 1° | 0.25° × 0.25° | 33/75 | LM4.0 | Simple bulk | Donner scheme | RRTM-G | K-profile |
| GFDL-ESM4* | ESM | NOAA-GFDL, USA | 1° × 1° | 0.25° × 0.25° | 49/75 | LM4.1 | Single-layer urban | Donner scheme | RRTM-G | K-profile |
| INM-CM4-8* | AOGCM | INM, Russia | 2° × 1.5° | 1° × 0.5° | 21/40 | Simple LSM | Bulk scheme | Betts-Miller-Janjic | Delta-Eddington | Monin-Obukhov |
| INM-CM5-0* | AOGCM | INM, Russia | 2° × 1.5° | 0.5° × 0.25° | 73/40 | Simple LSM | Bulk scheme | Betts-Miller-Janjic | Delta-Eddington | Monin-Obukhov |
| IPSL-CM6A-LR* | AOGCM | IPSL, France | 2.5° × 1.25° | 1° × 1° | 79/75 | ORCHIDEE | Simple urban | Emanuel scheme | RRTM-G | Mellor-Yamada |
| MIROC6* | AOGCM | JAMSTEC/AORI/NIES, Japan | T85 (~150km) | 1° × 1° | 81/63 | MATSIRO6 | Single-layer urban | Prognostic A-S | MSTRN | Mellor-Yamada |
| MPI-ESM1-2-HR* | ESM | MPI-M, Germany | T127 (~100km) | 0.4° × 0.4° | 95/40 | JSBACH3.2 | TERRA_URB | Tiedtke-Nordeng | RRTM-G | TKE-based |
| MPI-ESM1-2-LR | ESM | MPI-M, Germany | T63 (~200km) | 1.5° × 1.5° | 47/40 | JSBACH3.2 | Simple bulk | Tiedtke-Nordeng | RRTM-G | TKE-based |
| MRI-ESM2-0* | ESM | MRI, Japan | TL159 (~120km) | 1° × 0.5° | 80/60 | HAL | SiBUC | Yoshimura scheme | MRI-RAD | Mellor-Yamada |
| NESM3 | AOGCM | NUIST, China | T63 (~200km) | 1° × 1° | 47/46 | CLM4.0 | No explicit scheme | Mass flux scheme | RRTM-G | HB |
| NorESM2-MM* | ESM | NCC, Norway | 1° × 1° | 1° × 1° | 32/70 | CLM5 | Multi-layer urban | Zhang-McFarlane | RRTM-G | UW-PBL |
| TaiESM1 | ESM | AS-RCEC, Taiwan | 1° × 1° | 1° × 1° | 30/60 | CLM4.0 | Simple bulk | Zhang-McFarlane | RRTM-G | HB |

**Abbreviations:**

- PBL: Planetary Boundary Layer
- TKE: Turbulent Kinetic Energy
- RRTM-G: Rapid Radiative Transfer Model for GCMs
- HB: Holtslag-Boville scheme
- YSU: Yonsei University scheme
- CAPE: Convective Available Potential Energy
- TEB: Town Energy Balance
- A-S: Arakawa-Schubert
- UW-PBL: University of Washington PBL scheme
- CBR: Cuxart-Bougeault-Redelsperger scheme

**Notes:**

1. Resolution:
   - Horizontal resolutions are given either in degrees or spectral truncation (T/TL numbers)
   - Ocean resolutions are in degrees latitude × longitude
2. Urban Scheme Classifications:
   - No explicit scheme: Urban areas treated as generic land surface
   - Simple bulk: Basic modification of rural surface parameters
   - Single-layer: Urban canopy represented as one layer
   - Multi-layer: Sophisticated representation with multiple vertical layers
   - TEB/TERRA_URB: Specific urban canopy models
3. Models with asterisks (*) exceeded the 725-point threshold in our analysis


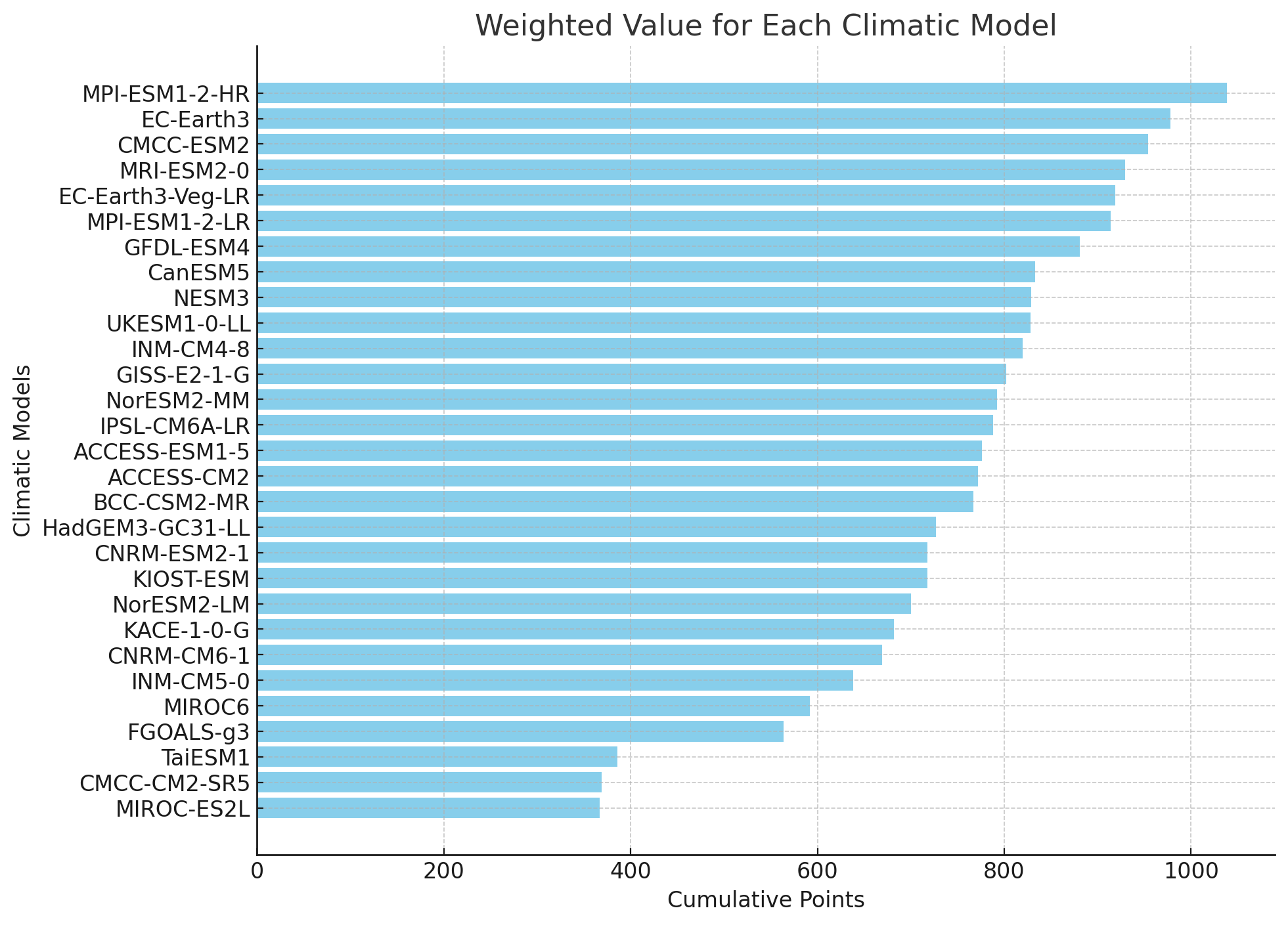


**Figure S2.** Comparison of heatwave indices from climate models and ERA5 reanalysis data based on the cumulative point. Higher value indicates better agreement between climate model simulations and the benchmark ERA5 reanalysis dataset.

**
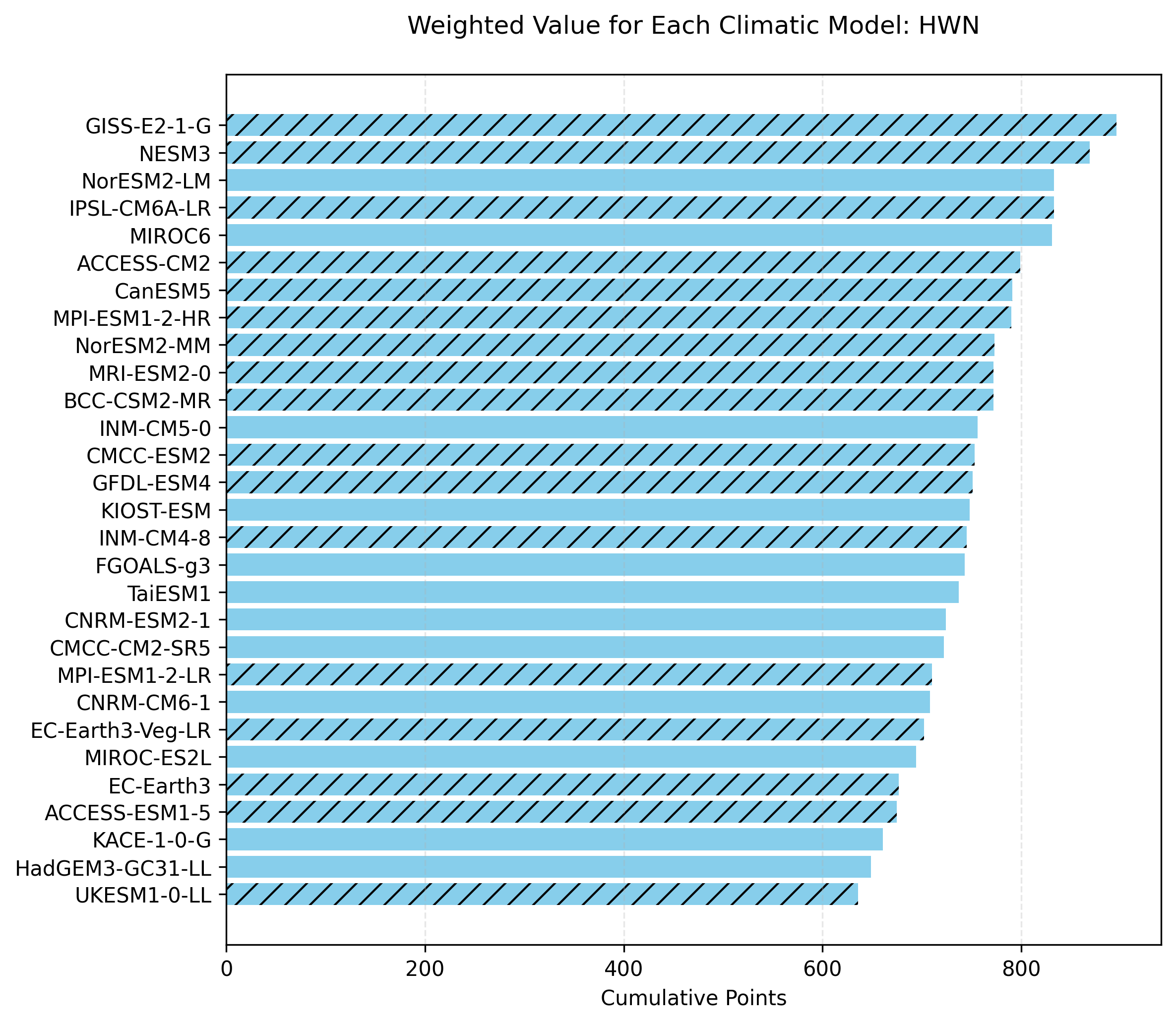
**

**Figure S3.** Quantitative assessment of climate models in simulating Heatwave Number (HWN) compared against ERA5 reanalysis data. The cumulative points reflect the models' capacity to accurately represent the frequency of heatwave events across the study region.


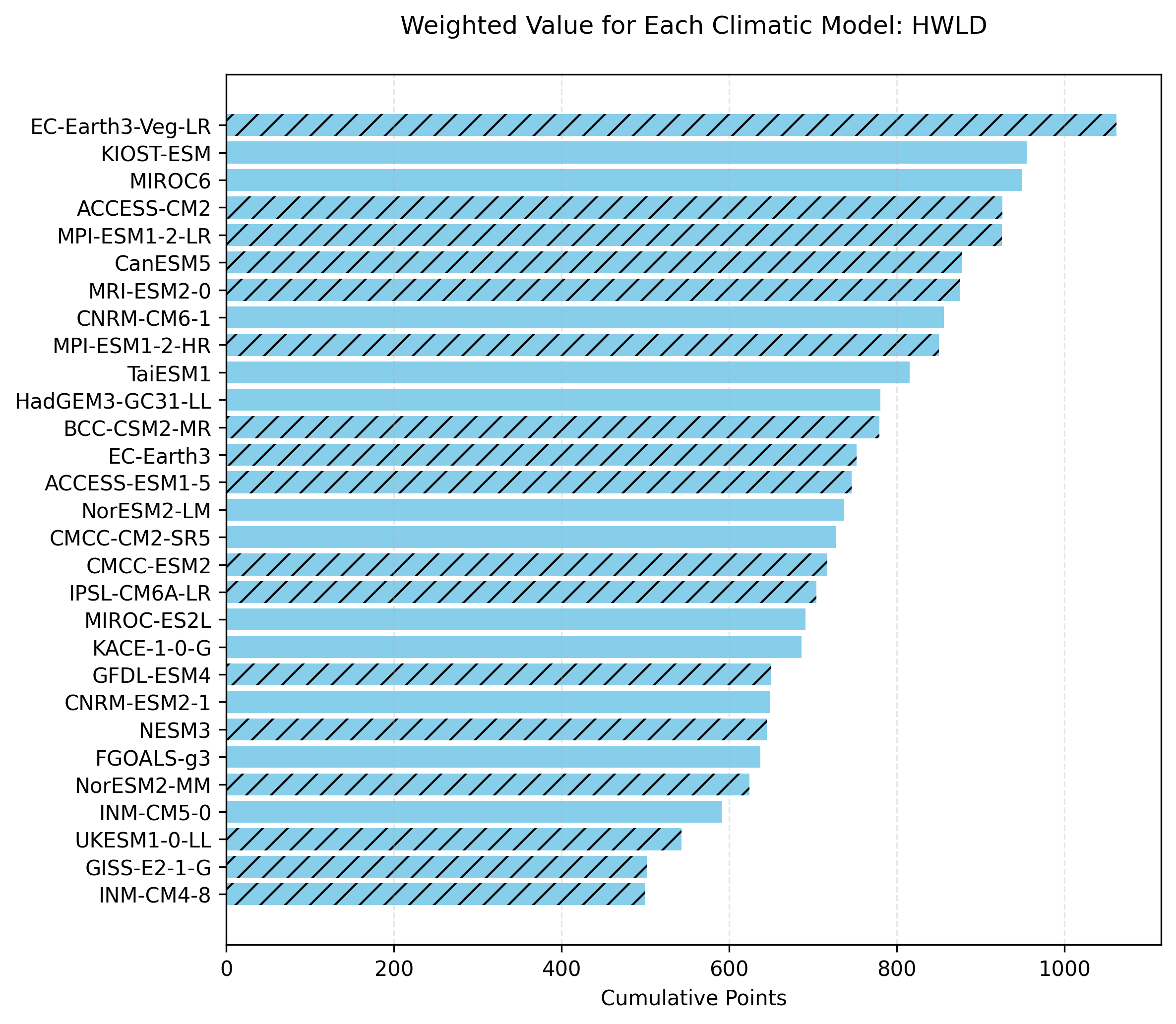


**Figure S4.** Comparative analysis of Heatwave Length Duration (HWLD) simulations across climate models, benchmarked against ERA5 reanalysis data. The ranking indicates each model's ability to capture the persistence of individual heatwave events.


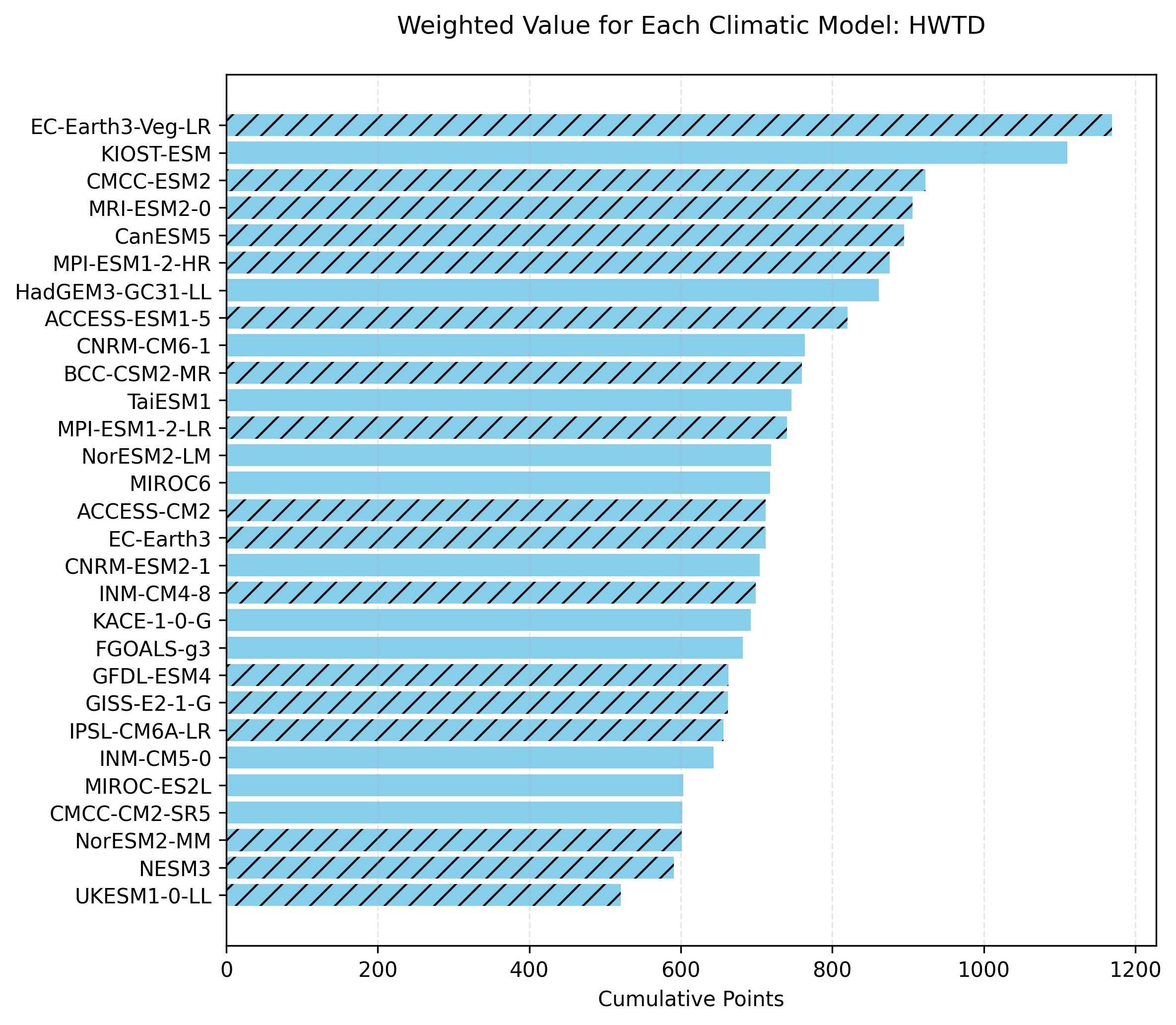


**Figure S5.** Evaluation of climate models' performance in simulating Heatwave Total Duration (HWTD), measured against ERA5 reanalysis data. This metric assesses the models' capability to represent the cumulative duration of heatwave conditions.


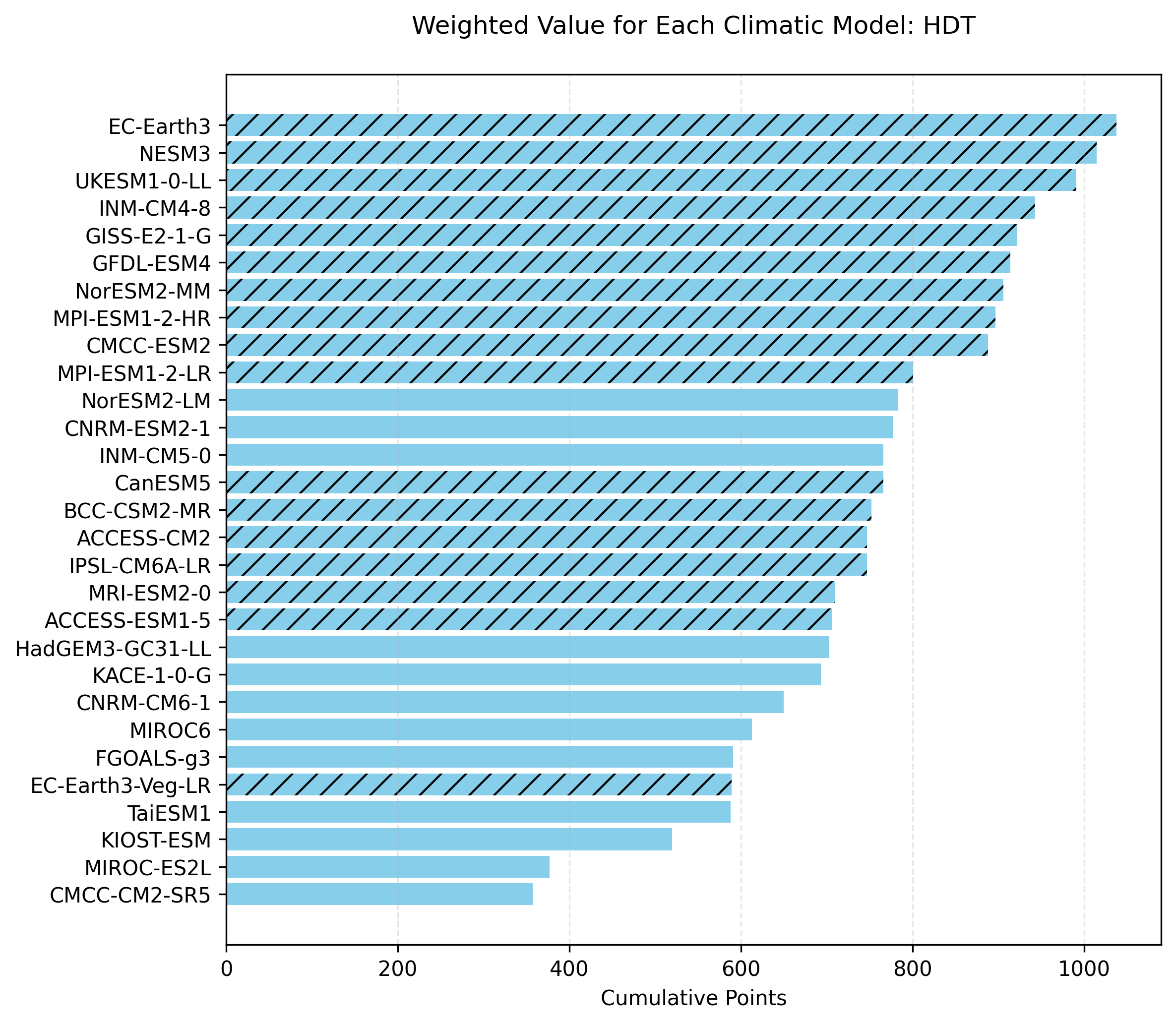


**Figure S6.** Assessment of Hot Days Threshold (HDT) representations across climate models, compared with ERA5 reanalysis data. The rankings reflect each model's accuracy in capturing temperature threshold exceedances.


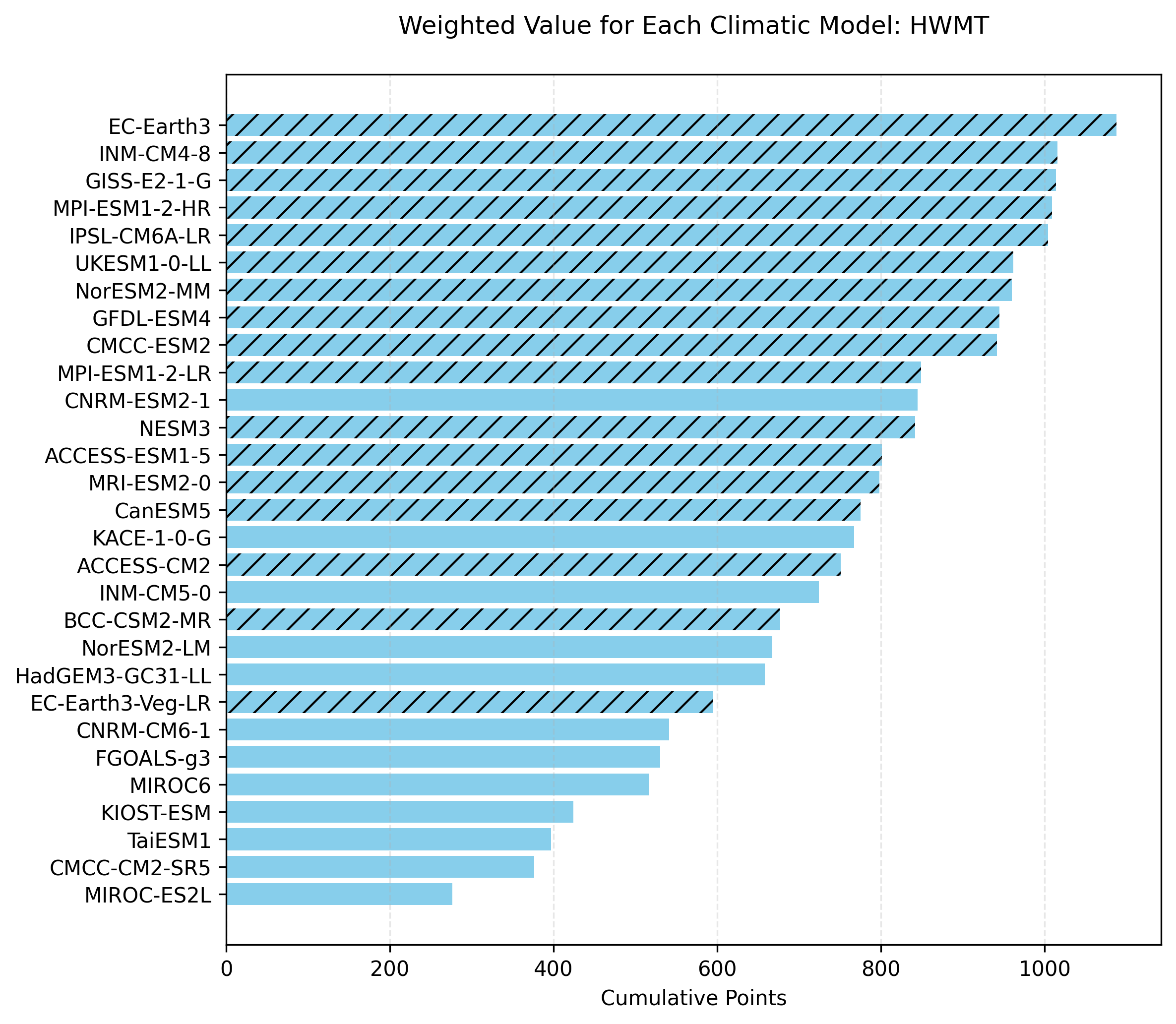


**Figure S7.** Analysis of climate models' fidelity in simulating Heatwave Mean Temperature (HWMT) patterns, validated against ERA5 reanalysis data. This metric evaluates the models' ability to represent the intensity of heatwave events.


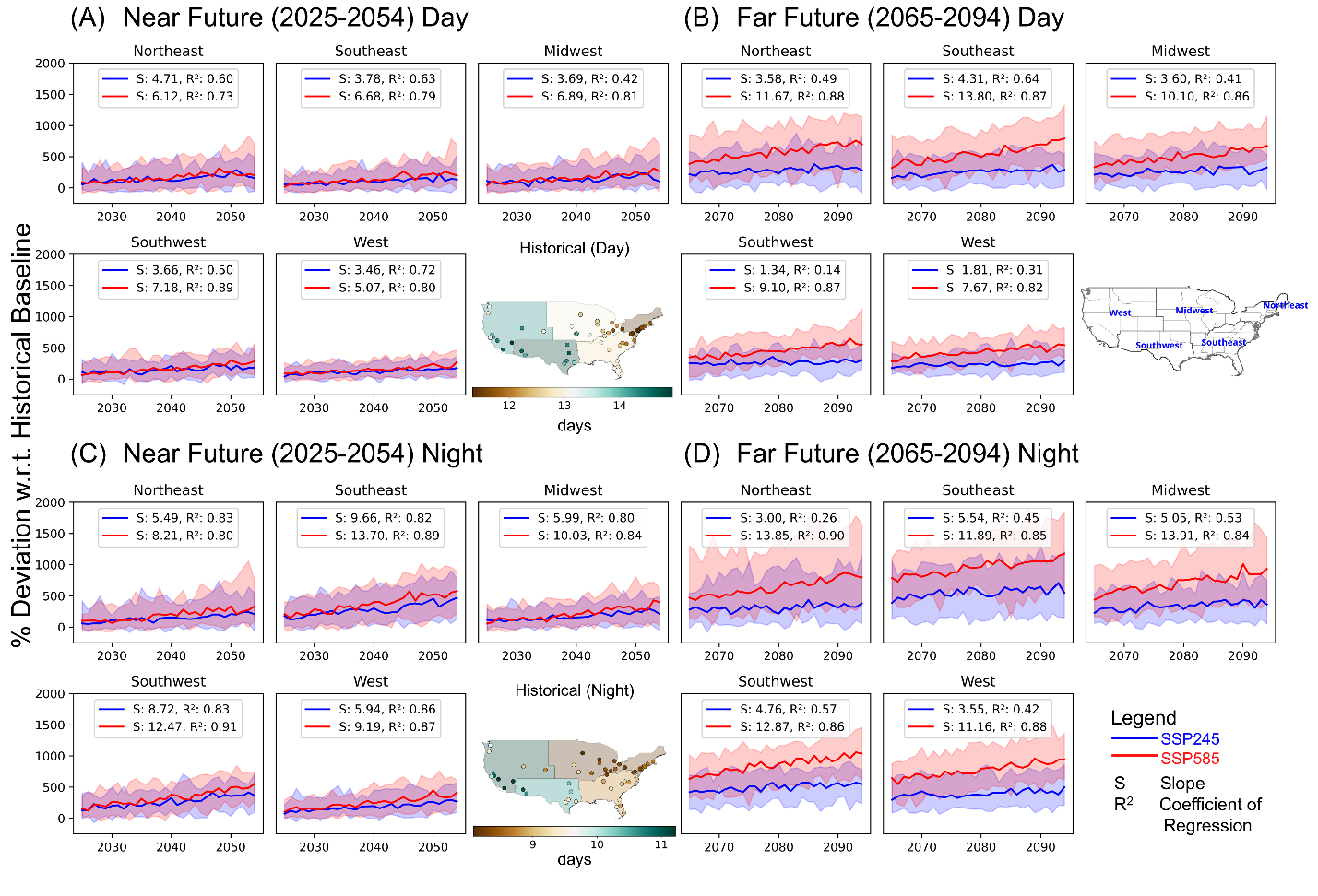


**Figure S8.** Projected percentage change in annual heatwave total duration (HWTD) for daytime (A, B) and nighttime (C, D) in the near future (2025-2054) and far future (2065-2094) relative to the baseline period (1985-2014) across different regions of the study area by an ensemble median of the heatwave total duration shown by 17 selected climate models. Percentage changes are calculated as ((future - baseline)/baseline) × 100. Shaded areas represent the interquartile range (25th to 75th percentile) across the climate models, indicating the spread in model projections. The spatial map in panel A shows the daytime HWTD during the historical baseline period, while the map in panel C shows the nighttime HWTD during the baseline. Line graphs at panels A and C display the projections for the near future, while panels B and D show the far future projections. The blue line represents the SSP2-4.5 scenario, and the red line represents the SSP5-8.5 scenario, with corresponding slope and R² values shown for each region to quantify the strength of temporal trends. Positive values indicate an increase in HWN while negative values indicate a decrease.


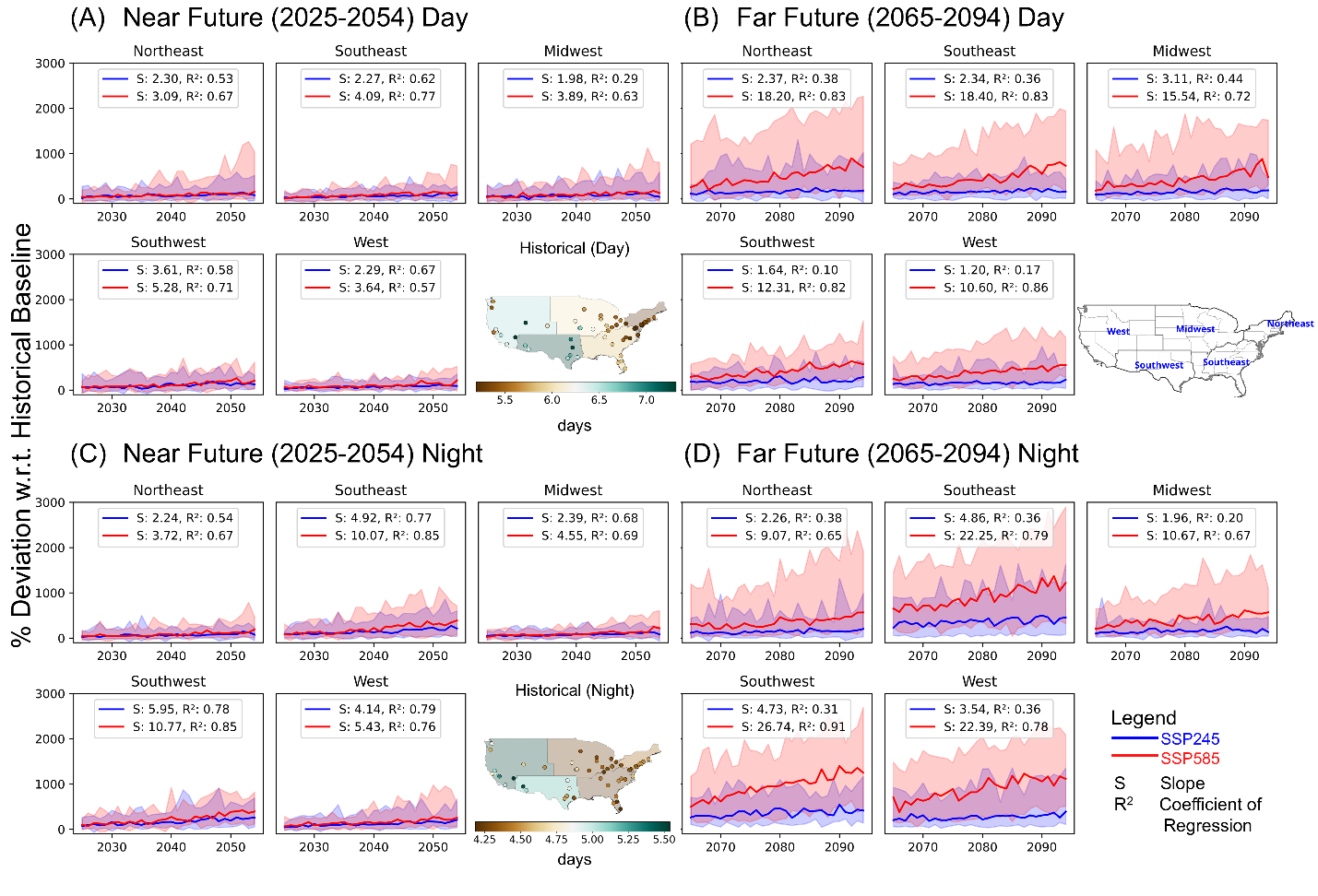


**Figure S9.** Projected percentage change in annual heatwave longest duration (HWLD) for daytime (A, B) and nighttime (C, D) in the near future (2025-2054) and far future (2065-2094) relative to the baseline period (1985-2014) across different regions of the study area by an ensemble median of the heatwave longest duration shown by 17 selected climate models. Percentage changes are calculated as ((future - baseline)/baseline) × 100. Shaded areas represent the interquartile range (25th to 75th percentile) across the climate models, indicating the spread in model projections. The spatial map in panel A shows the daytime HWLD during the historical baseline period, while the map in panel C shows the nighttime HWLD during the baseline. Line graphs at panels A and C display the projections for the near future, while panels B and D show the far future projections. The blue line represents the SSP2-4.5 scenario, and the red line represents the SSP5-8.5 scenario, with corresponding slope and R² values shown for each region to quantify the strength of temporal trends. Positive values indicate an increase in HWN while negative values indicate a decrease.


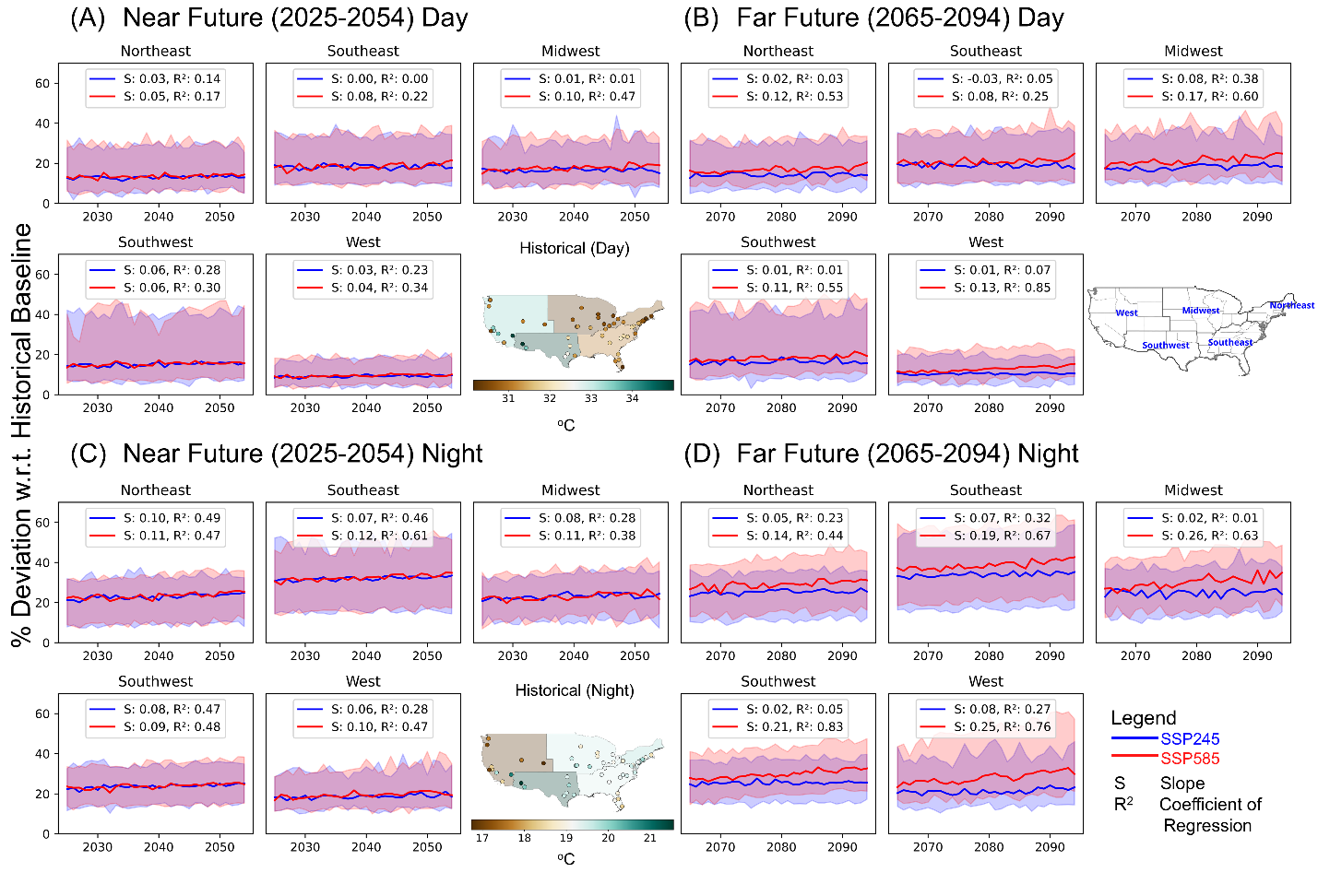


**Figure S10.** Projected percentage change in annual heatwave mean temperature (HWMT) for daytime (A, B) and nighttime (C, D) in the near future (2025-2054) and far future (2065-2094) relative to the baseline period (1985-2014) across different regions of the study area by an ensemble median of the heatwave mean temperature shown by 17 selected climate models. Percentage changes are calculated as ((future - baseline)/baseline) × 100. Shaded areas represent the interquartile range (25th to 75th percentile) across the climate models, indicating the spread in model projections. The spatial map in panel A shows the daytime HWMT during the historical baseline period, while the map in panel C shows the nighttime HWMT during the baseline. Line graphs at panels A and C display the projections for the near future, while panels B and D show the far future projections. The blue line represents the SSP2-4.5 scenario, and the red line represents the SSP5-8.5 scenario, with corresponding slope and R² values shown for each region to quantify the strength of temporal trends. Positive values indicate an increase in HWN while negative values indicate a decrease.


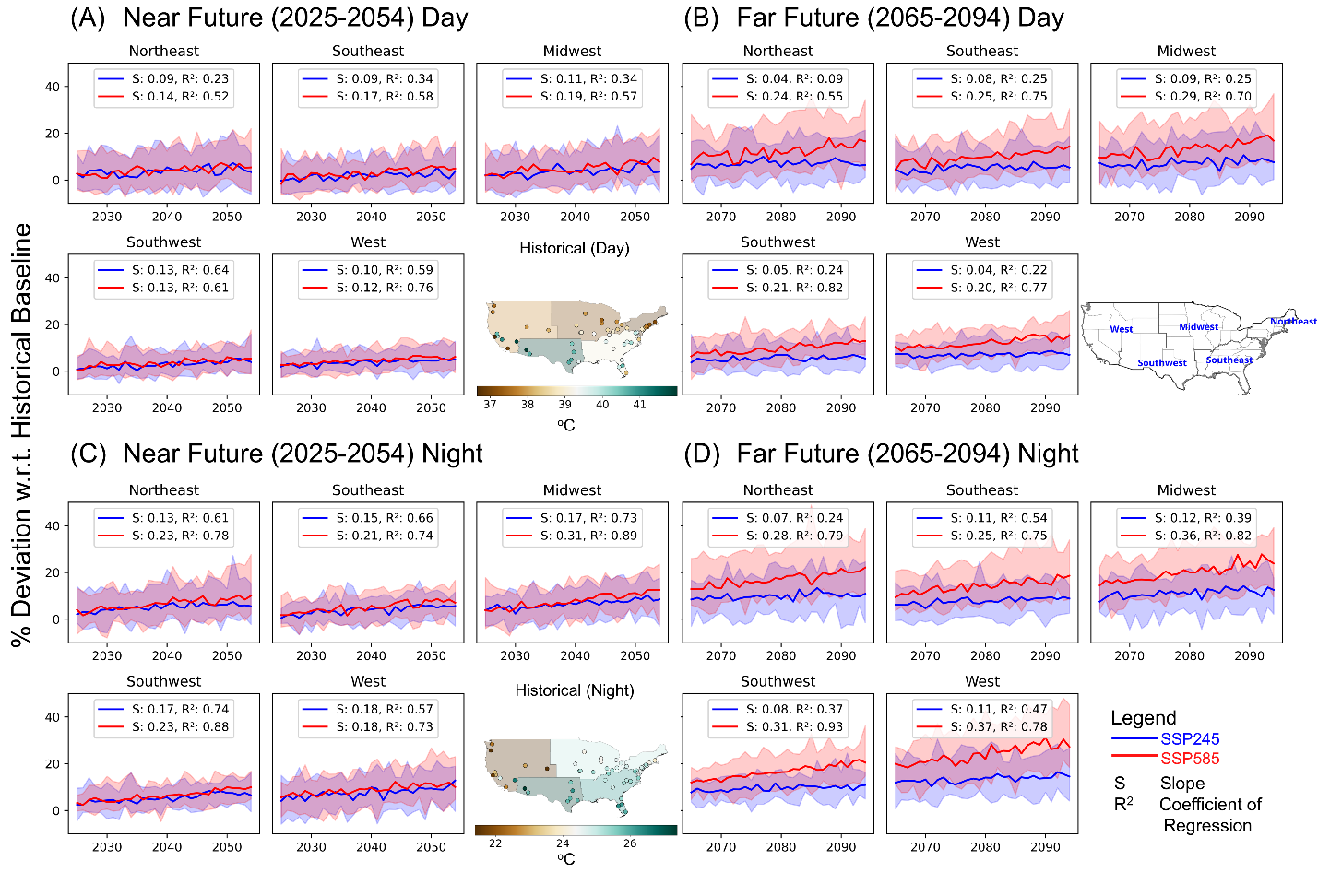


**Figure S11.** Projected percentage change in annual Hottest Heatwave Temperature (HDT/HHT) for daytime (A, B) and nighttime (C, D) in the near future (2025-2054) and far future (2065-2094) relative to the baseline period (1985-2014) across different regions of the study area by an ensemble median of the hottest heatwave temperature shown by 17 selected climate models. Percentage changes are calculated as ((future - baseline)/baseline) × 100. Shaded areas represent the interquartile range (25th to 75th percentile) across the climate models, indicating the spread in model projections. The spatial map in panel A shows the daytime HDT/HHT during the historical baseline period, while the map in panel C shows the nighttime HDT/HHT during the baseline. Line graphs at panels A and C display the projections for the near future, while panels B and D show the far future projections. The blue line represents the SSP2-4.5 scenario, and the red line represents the SSP5-8.5 scenario, with corresponding slope and R² values shown for each region to quantify the strength of temporal trends. Positive values indicate an increase in HWN while negative values indicate a decrease.


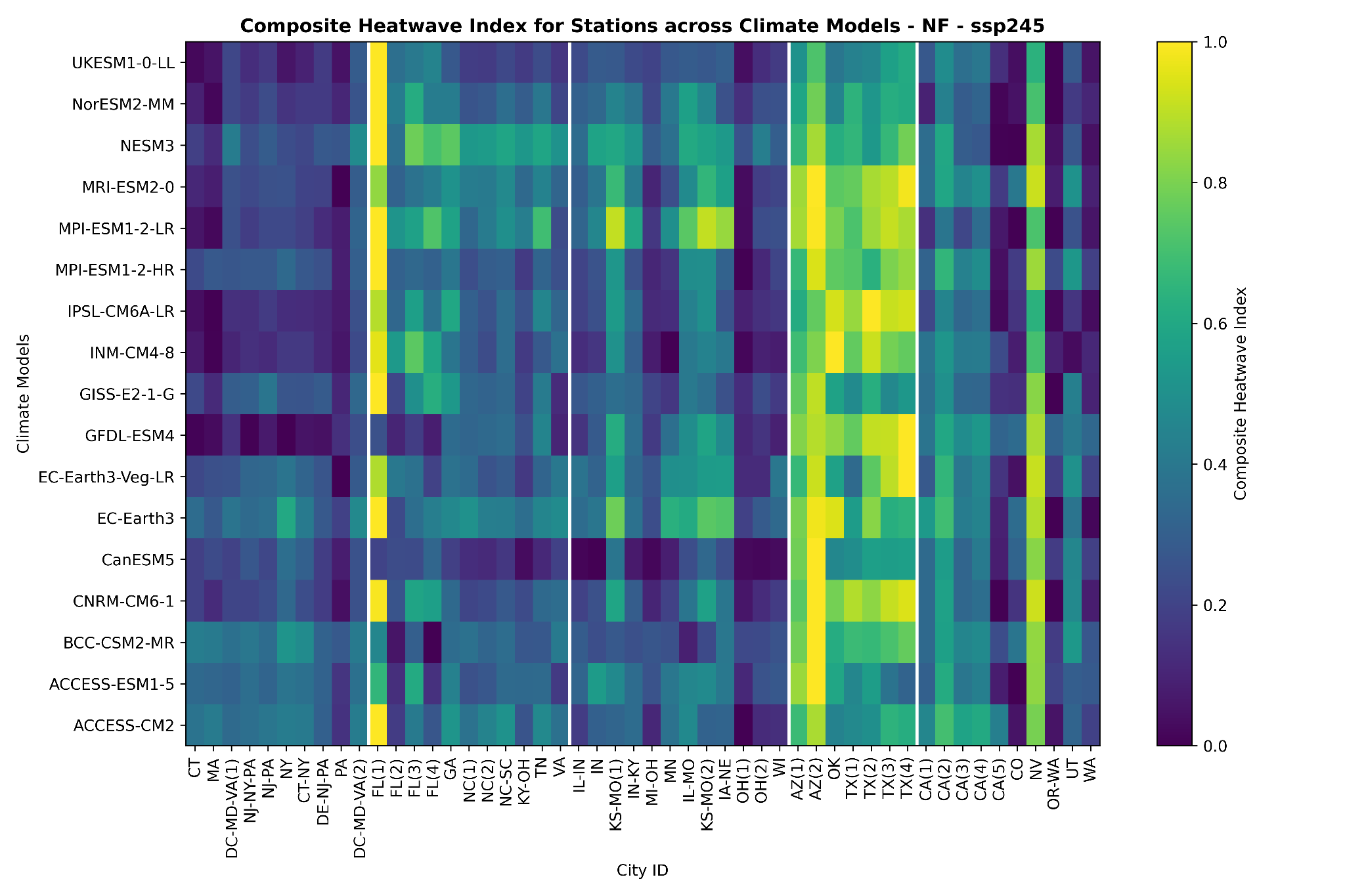


**Figure S12:** The composite heatwave index for different cities as simulated by the 17 selected CMIP6 global climate models during the 2025-2054 Near Future baseline period at SSP2-4.5 scenario. Cities are grouped into the broader Northeast, Southeast, Midwest, Southwest, and West regions delineated by white lines. Refer to the Supplementary Data table for decoding the city ID to the corresponding names. Higher index values indicate locations facing more intense, frequent, and enduring historical heatwaves based on the model SSP2-4.5 reanalysis.


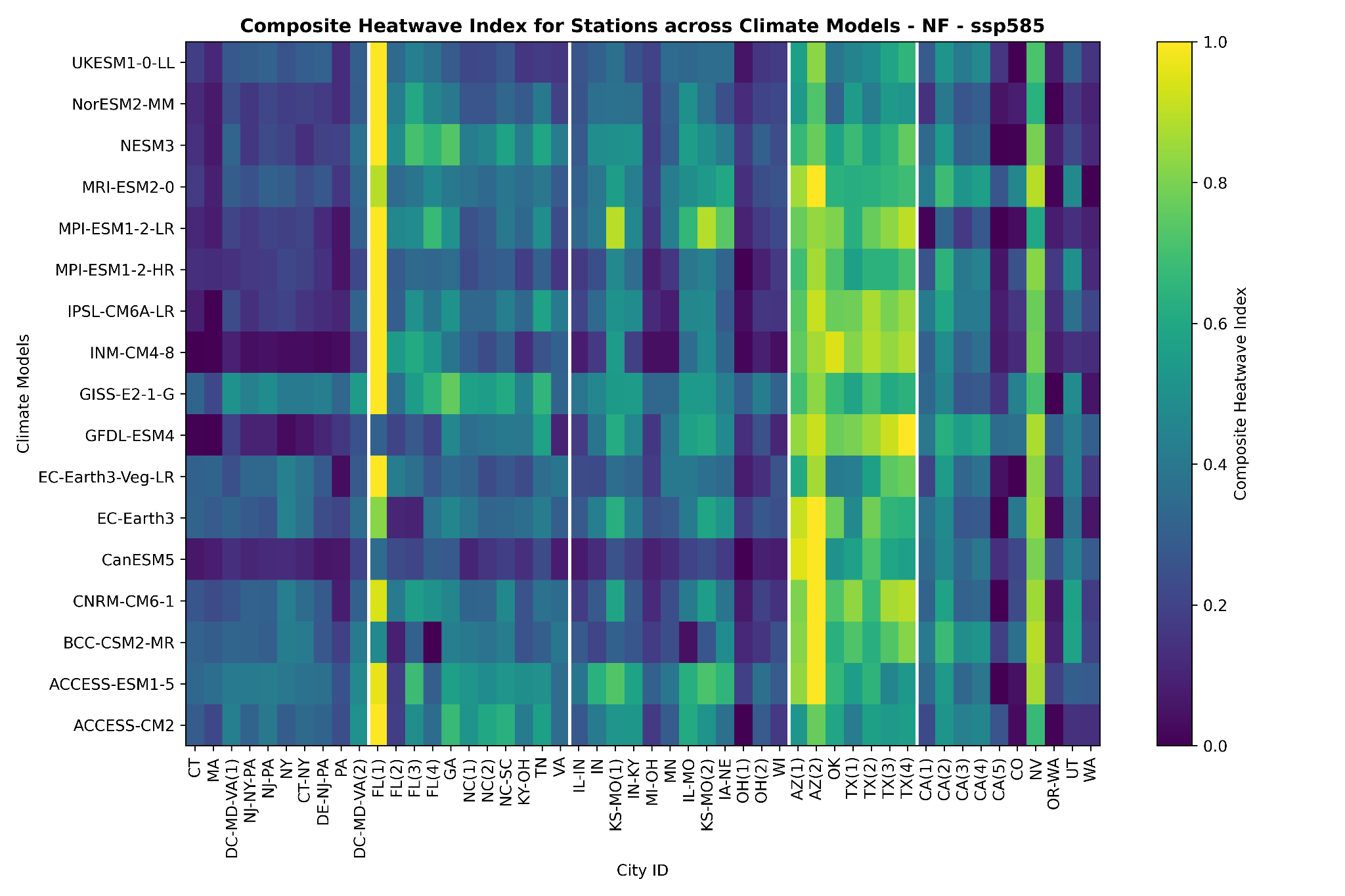


**Figure S13:** The composite heatwave index for different cities as simulated by the 17 selected CMIP6 global climate models during the 2025-2054 Near Future baseline period under SSP5-8.5. Cities are grouped into the broader Northeast, Southeast, Midwest, Southwest, and West regions delineated by white lines. Refer to the Supplementary Data table for decoding the city ID to the corresponding names. Higher index values indicate locations facing more intense, frequent, and enduring historical heatwaves based on the model SSP5-8.5 reanalysis.


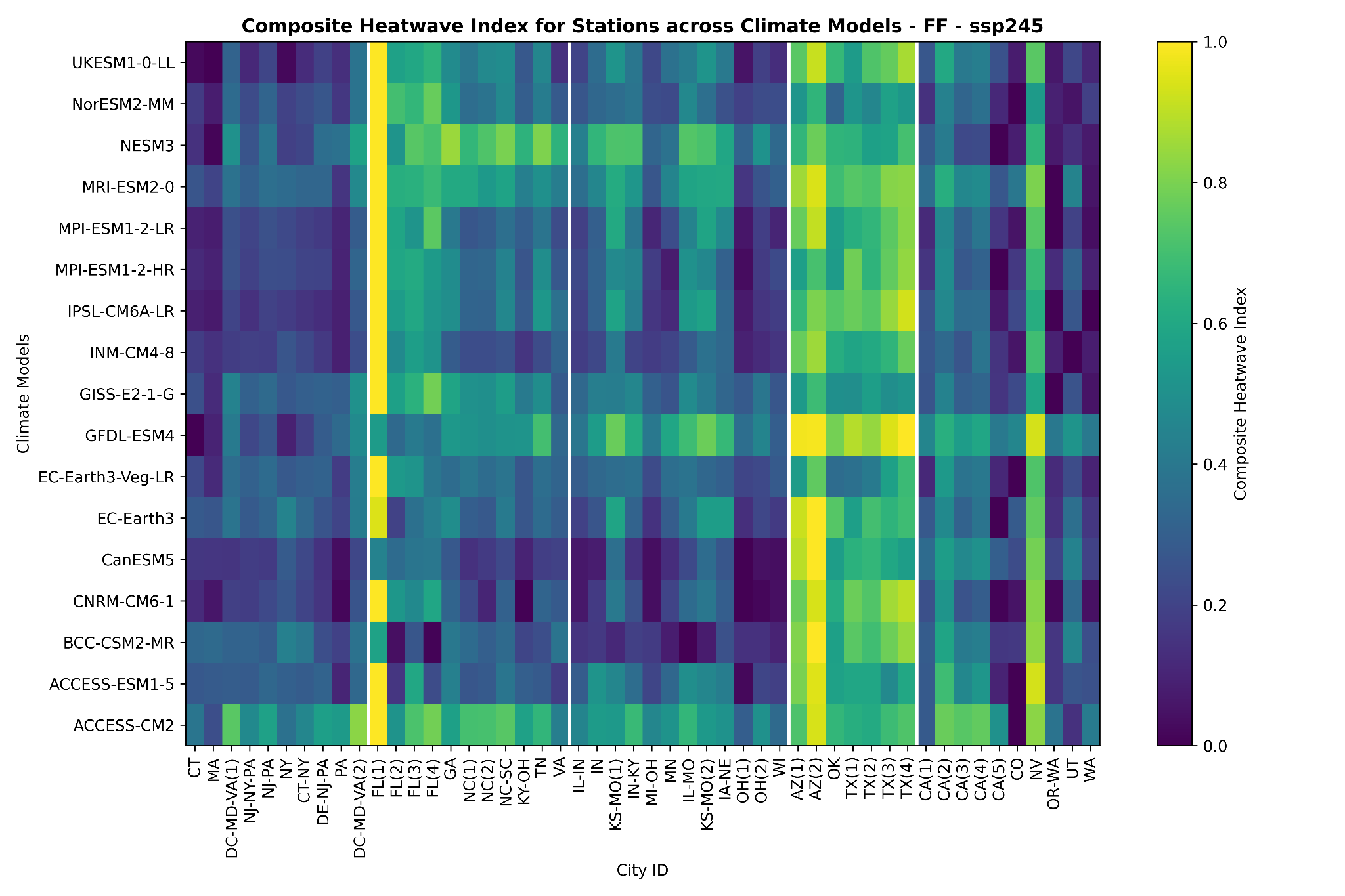


**Figure S14:** The composite heatwave index for different cities as simulated by the 17 selected CMIP6 global climate models during the 2065-2094 Far Future baseline period under SSP2-4.5. Cities are grouped into the broader Northeast, Southeast, Midwest, Southwest, and West regions delineated by white lines. Refer to the Supplementary Data table for decoding the city ID to the corresponding names. Higher index values indicate locations facing more intense, frequent, and enduring historical heatwaves based on the model SSP2-4.5 reanalysis.


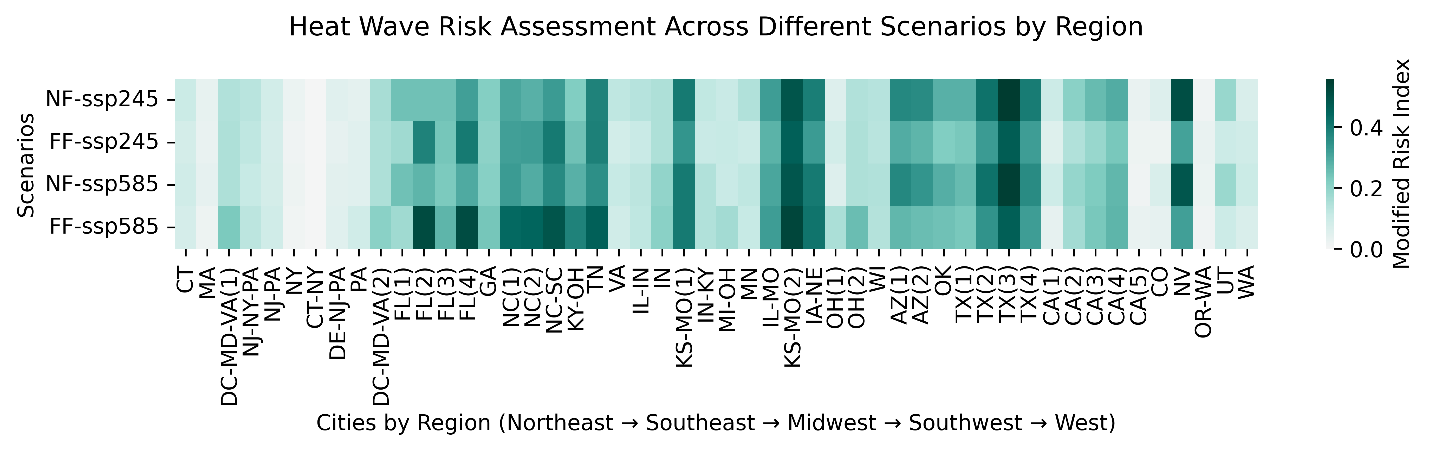


Figure **S15**: Heatmap of Modified Heatwave Risk by City ID. This figure visualizes the spatial distribution of modified heatwave risk across urban areas in the United States. The risk index is derived as the product of the composite heatwave index (normalized between 0 and 1) and the population change w.r.t historical timeframe (also normalized), highlighting regions where extreme heat events and high population change contribute to increased vulnerability. Darker shades indicate areas of higher risk.


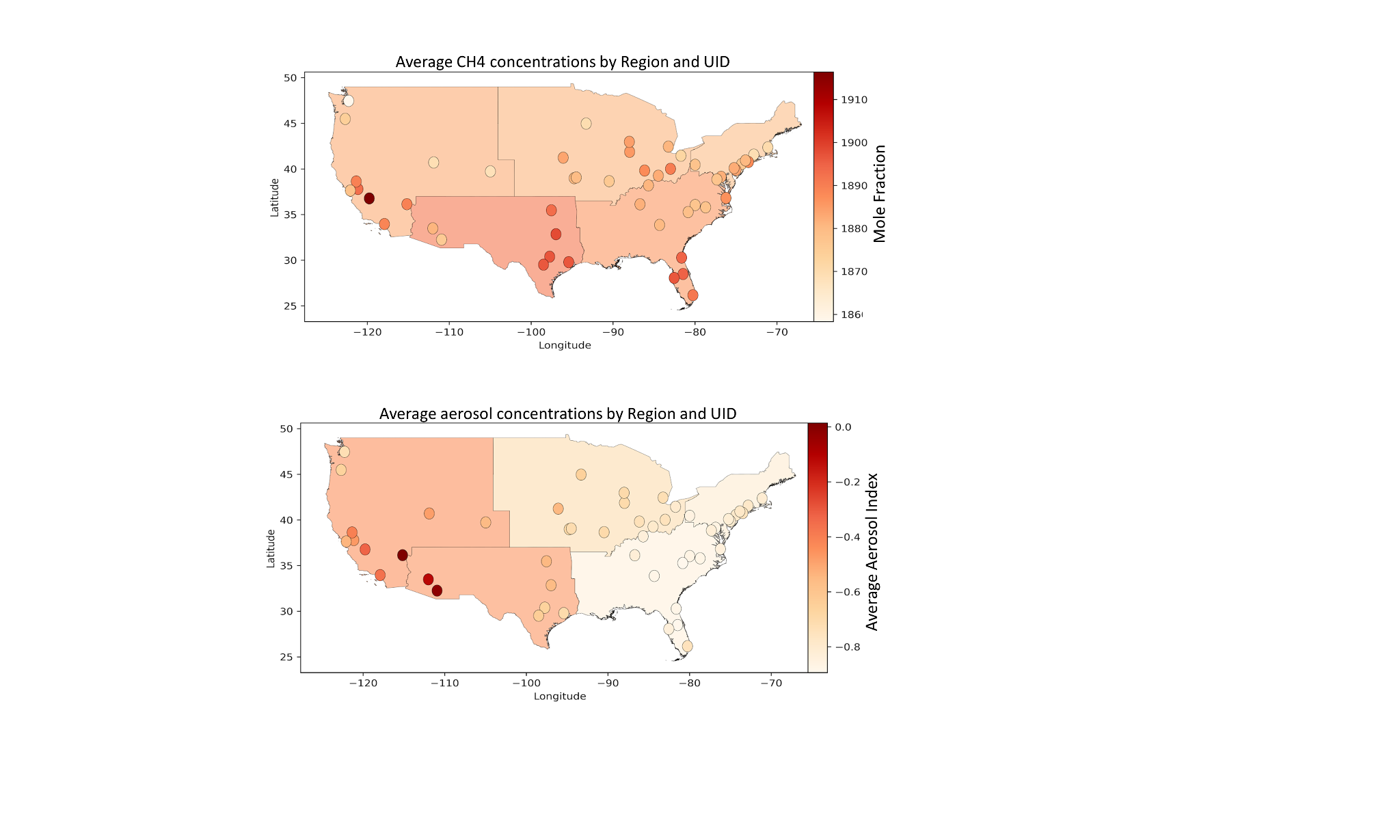


**Figure S16:** Maps of Methane (CH4) and Aerosol Index in US Cities and Regions: Data from TROPOspheric Monitoring Instrument (TROPOMI) aboard the Sentinel-5P satellite, showcasing variations in CH4 and aerosol levels across different urban and regional areas in the US.


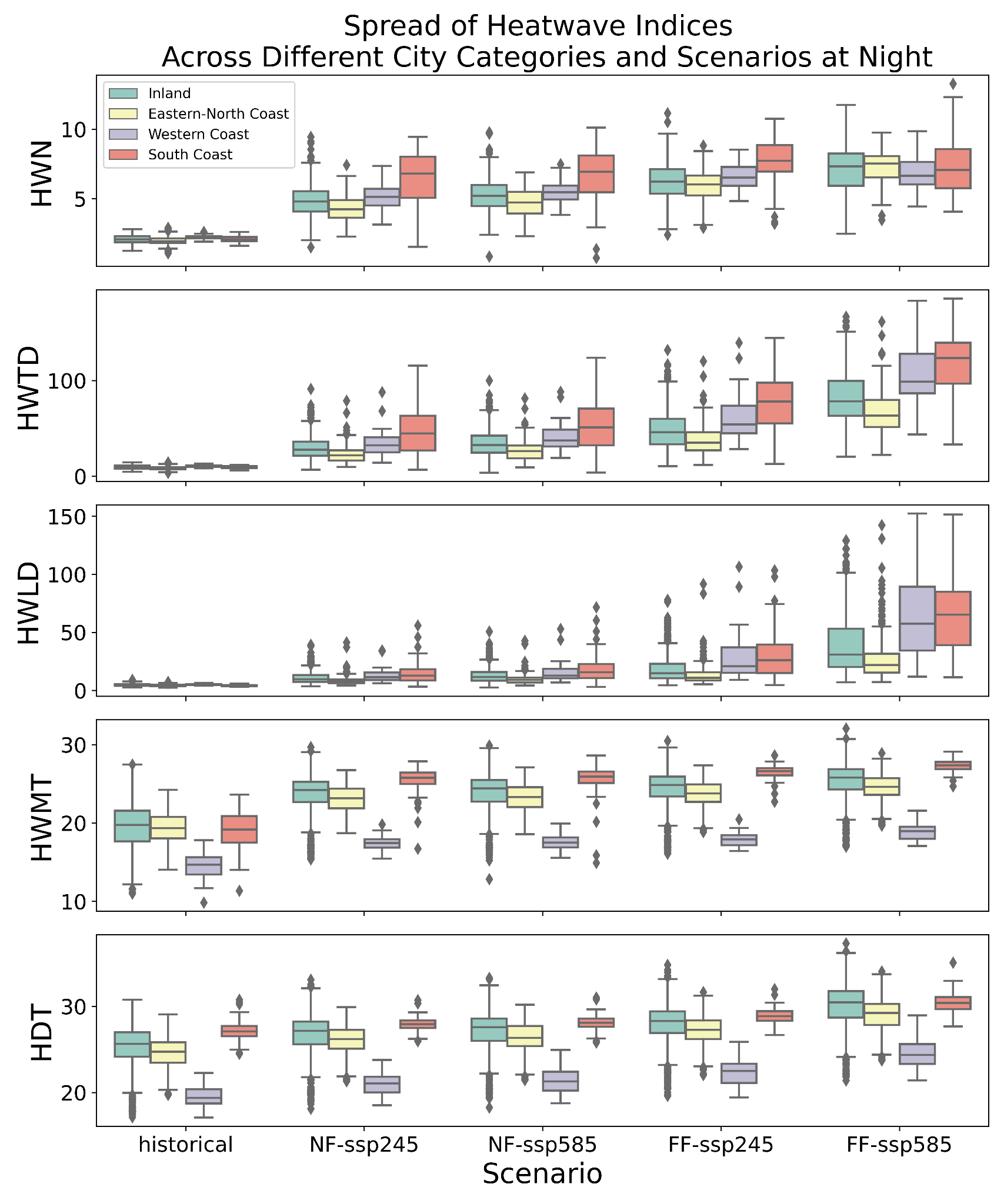


**Figure S17.** Box plots illustrating the heatwave characteristics, including frequency (HWN), duration (HWTD and HWLD), and intensity (HWMT and HDT), across different scenarios and regions at night. The scenarios include historical (baseline), near-future with moderate (NF-SSP2-4.5) and high (NF-SSP5-8.5) greenhouse gas concentrations, and far-future with moderate (FF-SSP2-4.5) and high (FF-SSP5-8.5) greenhouse gas concentrations. The regions comprise the Western Coast, South Coast, East-North Coast, and inland areas. The box plots enable visual comparison and identification of patterns in heatwave characteristics among coastal and inland regions under various climate scenarios.
